# The Spatial and Temporal Distribution of Dissolved Organic Carbon Exported from Three Chinese Rivers to the China Sea

**DOI:** 10.1371/journal.pone.0165039

**Published:** 2016-10-18

**Authors:** Guohua Shi, Changhui Peng, Meng Wang, Shengwei Shi, Yanzheng Yang, Junyao Chu, Junjun Zhang, Guanghui Lin, Yan Shen, Qiuan Zhu

**Affiliations:** 1 Laboratory for Ecological Forecasting and Global Change, College of Forestry, Northwest A&F University, Yangling, Shaanxi, 712100, China; 2 Department of Biology Sciences, Institute of Environment Sciences, University of Quebec at Montreal, C.P. 8888, Succ. Centre-Ville, Montreal, H3C 3P8, Canada; 3 Ministry of Education Key Laboratory for Earth System Modeling, Center for Earth System Science, Tsinghua University, Beijing, 100084, China; 4 Key Laboratory of Ecosystem Network Observation and Modeling, Institute of Geographic Sciences and Natural Resources Research, CAS, Beijing, 100101, China; 5 Institute of Metrological Administration and Strategic Planning, National Institute of Metrology, AQSIQ, Beijing, 100013, China; 6 Faculty of Life Science and Technology, Central South University of Forestry and Technology, Changsha, Hunan, 410004, China; Old Dominion University, UNITED STATES

## Abstract

The lateral transport of dissolved organic carbon (DOC) plays an important role in linking the carbon cycles of terrestrial and aquatic ecosystems. Neglecting the lateral flow of dissolved organic carbon can lead to an underestimation of the organic carbon budget of terrestrial ecosystems. It is thus necessary to integrate DOC concentrations and flux into carbon cycle models, particularly with regard to the development of models that are intended to directly link terrestrial and ocean carbon cycles. However, to achieve this goal, more accurate information is needed to better understand and predict DOC dynamics. In this study, we compiled an inclusive database of available data collected from the Yangtze River, Yellow River and Pearl River in China. The database is collected based on online literature survey and analysed by statistic method. Overall, our results revealed a positive correlation between DOC flux and discharge in all three rivers, whereas the DOC concentration was more strongly correlated with the regional net primary productivity (NPP). We estimated the total DOC flux exported by the three rivers into the China Sea to be approximately 2.73 Tg yr^-1^. Specifically, the annual flux of DOC from the Yangtze River, Yellow River and Pearl River was estimated to be 1.85 Tg yr^-1^, 0.06 Tg yr^-1^ and 0.82 Tg yr^-1^, respectively, and the average annual DOC concentrations were estimated to be 2.24 ± 0.53 mg L^-1^, 2.70 ± 0.38 mg L^-1^ and 1.51 ± 0.09 mg L^-1^, respectively. Seasonal variations in DOC concentrations are greatly influenced by the interaction between temperature and precipitation. NPP is significantly and positively related to the DOC concentration in the Yangtze River and the Pearl River. In addition, differences in climate and the productivity of the vegetation may influence both the flux and concentrations of DOC transported by the rivers and thus potentially affect estuarine geochemistry.

## 1. Introduction

Rivers, particularly large rivers, connect the two most important carbon pools, i.e., the terrestrial and the oceanic pools. Approximately 1 Pg (1 Pg = 10^15^g) of carbon is transported annually by rivers to the oceans, of which dissolved organic carbon (DOC) constitutes 0.22 Pg [1]. Ludwig et al. [2] revealed that the DOC flux was mainly a function of discharge, basin slope, and the carbon content of the soils in the drainage basin, and the researchers estimated an annual riverine DOC flux to the coastal oceans of 0.21 Pg C yr^-1^. A more recent assessment by Dai et al. [3], who used the DOC concentrations in 118 world rivers and long-term average river discharges, estimates that the DOC flux is 0.17 Pg C yr^-1^ from the global river. The total amount of organic carbon transported by global rivers accounts for approximately 17% of the net carbon accumulation on continents or in oceans [4], which suggests that riverine organic carbon transportation plays an important role in the global carbon cycle. Therefore, to predict the feedback between the future climate and the global carbon cycle, it is necessary to understand the relationship between organic carbon transportation and climate. Although extensive research has been conducted on the world’s rivers [5, 6, 7, 8], it is still important to investigate the influences of climate factors on organic carbon transport at a regional scale because our knowledge of the mechanisms driving the temporal and spatial variations in carbon transportation is limited. Moreover, the differences in the environmental characteristics between river drainage basins are so enormous that such an analysis must be undertaken independently for each river drainage [9].

The export of carbon to the oceans by Chinese rivers is estimated to range from 0.038–0.070 Pgyr^-1^[10]. Specifically, many previous studies have focused on the organic carbon flux and the spatiotemporal variation of DOC transported in the Pearl River [11,12,13]; the temporal characteristics of organic carbon in the mainstream Yangtze River and the effect of the Three Gorges Dam on organic carbon flux [14,15,16]; the effect of human activities and soil erosion that drove the burial of organic carbon and carbon output in the Yellow River basin [17,18]; and the temporal factors influencing organic carbon transport in the Yellow River estuary [19, 20]. However, only the individual components of carbon flux have been studied in these rivers, and thus an estimate of the total organic carbon flux in Chinese rivers is still necessary. At the same time, due to low sampling frequency and strong seasonal variations in flow, sediment and water chemistry for most rivers [21], the accuracy of previous estimates has been called into question. It is therefore necessary to integrate the various estimates of organic carbon flux exported by Chinese rivers into the ocean. In addition, Ying et al. [22] showed that there is a positive linear relationship between the soil organic carbon (SOC) and DOC concentrations in subtropical regions. The majority of variation in DOC concentrations in European forest soils correlates with NH_4_^+^, C/N, Al, and Fe as the most important predictors.DOC concentrations were also generally higher in very acidic soils (pH<4.2) than in more basic soils [23]. The terrestrial export of DOC depends on the net primary production (NPP) of the terrestrial system, plant material degradation to DOC in soils [24] and hydrological flushing of this DOC to surface waters [25, 26]. This suggested that the concentration of DOC varies in different soils and climate across large spatial scales, soil types and vegetation covers. Expanding the scale of research from individual components to the whole river basin is expected to improve our understanding of the environmental controls on organic carbon transport. It should also be helpful in terms of achieving an accurate estimation of the flux in global organic carbon export.

In this paper, we examine the variations in flux and concentrations of DOC transported by three major rivers in China—the Yellow River, Yangtze River and Pearl River. We utilized the most up-to-date literature in the DOC field to estimate the DOC flux exported from these three rivers to the oceans. We explore the spatial and temporal variations in the distribution of DOC and examine the influence that factors such as hydrology, net primary productivity (NPP) and climate conditions exert on DOC concentrations. The objectives of this study areas follows: 1) identify potential environmental factors controlling the DOC concentrations and the flux from terrestrial to aquatic systems and 2) compile all of the available DOC data to quantify the budget of the export from three Chinese rivers of DOC flux to estuaries. (3) analyze the spatial and seasonal distribution of DOC exported from three Chinese rivers to the China Sea.

## 2. Data Sources and Methods

### 2.1 Study area

**Fig 1:**
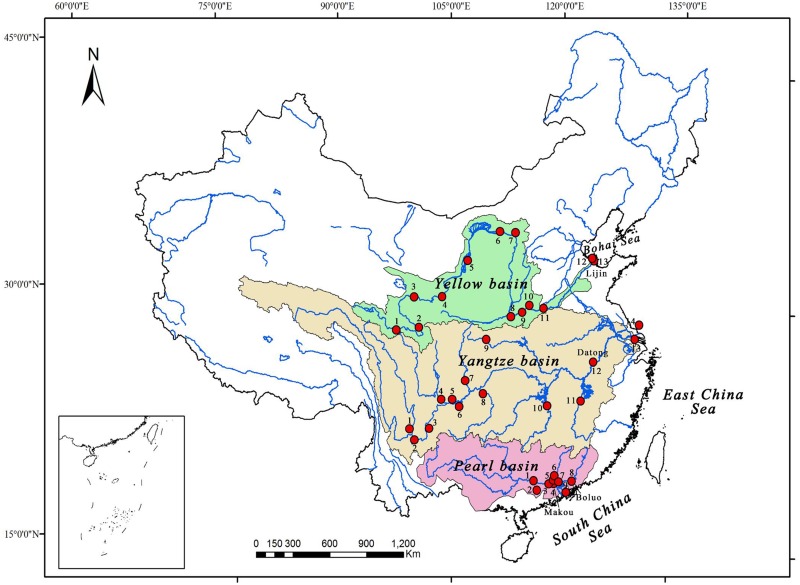
Spatial distribution of DOC from the three river basins in China. The different background colours represent the Yellow, Yangtze and Pearl River basins, respectively. Red solid circle represents the location of the sampling stations in the three river basins. The description of the serial number of each site used in this analysis can be found in the S2 Table.

The Yellow, Yangtze, and Pearl River basins account for approximately 80% of the drainage area in China. Moreover, three rivers are the most of drainage areas from river to ocean, and there is more research works conducted about terrestrial carbon cycling than others’ in China. Specifically, The Yellow River originates in the eastern Qinghai–Tibetan Plateau and flows into the Bohai Sea [27]. The Loess Plateau, which largely overlaps with the middle Yellow River basin, is the major sediment source for the Yellow River [28]. An important feature of the Yellow River is that it has been recognized as one of the highest sediment Load Rivers in the world [29]. The Yangtze River originates in the Qinghai-Tibetan plateau and discharges to the East China Sea. The low reach of the Yangtze River passes through a temperate climate region in southeast China, where the terrestrial vegetation coverage is relatively abundant. The Pearl River is fed by the Xijiang, Beijiang, and Dongjiang. The Xijiang is the main stem of the Pearl River, accounting for 77.8% and 63.9% of the drainage area and annual discharge of the Pearl River, respectively [30]. The main characteristics of three rivers are shown in Table 1, and their location is shown in Fig 1.

**Table 1:** Characteristics of three rivers in China.

River	Longitude	Latitude	Drainage area km^2^	Total length km	Sediment loads 10^6^ g yr^-1^	Annual dischargekm^3^ yr^-1^	Annual temperature °C	Annual precipitation mm	Climate zone
Yellow River	90°33'–122°25'E	24°30'–35°45'N	752,443	5,464	1 × 10^9^[29]	15	8–14[28]	476[28]	arid-semiarid
Yangtze River	95°53'–119°10'E	32°10'–41°50'N	1,800,000	6,300	0.5× 10^9^[27]	960	^/^	1100[16]	Subtropical monsoon
Pearl River	102°15'–115°35' E	21°50'–26°48'N	450,000	2,214	8.87× 10^7^	350	14–22[31]	1200–2200[31]	subtropical to tropical monsoon

^“/”^ represent no data

### 2.2 Data sources

#### 2.2.1 Sources for DOC and discharge data

Literature searches were performed using the online databases available through the Web of Science and the Chinese Academy of Sciences, with search terms mainly included “dissolved organic carbon”, “river” and “China”. The database collected screening criteria as follows: firstly, the data collected from measured data in hydro station, not including an incubation test. Secondly, the data collected from the mainstream and tributaries of three major rivers, not including soil solution DOC. We collected all relevant literature, including journal articles and dissertations, published in either English or Chinese. Few data were available from continuous long-term experimental sites (>3 years) that investigated DOC dynamics in China. All data on river discharge were based either on the water resources bulletin published by the Chinese government (http://www.mwr.gov.cn/zwzc/hygb/zghlnsgb/) or on the references mentioned above. In addition, the coordinates of the river mouths were identified using Google Maps. We collected the DOC database based on the literatures from 36 field sites in three rivers (shown in Fig 1). The general description of the sites and DOC concentrations used in this analysis can be found in the S2 Table.

#### 2.2.2 Vector database sources and treatment

Meteorological data from 2000 to 2013, provided by China meteorological science data sharing service (http://www.escience.gov.cn/metdata/page/index.html), was interpolated into 10 km resolution using ANUSPLIN [32], and the monthly temperature and precipitation in each regional catchment was calculated using ArcGIS. Annual NPP from 2000 to 2013, at a 1-km spatial resolution, was calculated via algorithms (MOD17A2/A3) [33].

### 2.3 Statistical methods and river network extraction

Due to the variations in the measurement units used in the databases, we used the formula below to unify the data on DOC flux:

1. The DOC export flux (*F*_*DOC*_) was calculated by the following equation:
  $$F_{DOC}=\overset{365}{\underset{i=1}{\sum }}C_{i}\times Q_{i}$$
  Where *F*_*DOC*_ represents the annual flux of DOC (Tg yr^-1^) and *C*_*i*_ and *Q*_*i*_ are the measured daily DOC concentration (mg L^-1^) and daily discharge (m^3^ d^-1^) at the site of data collection, respectively [11]. Specifically, *F*_*DOC*_ (Tg yr^-1^) = *C*_*i*_ (mg L^-1^) × *Q*_*i*_ (m^3^ d^-1^) × 365 = *C*_*i*_ (mg /1000 m^3^) × *Q*_*i*_ (m^3^ d^-1^) × 365 = *C*_*i*_ × *Q*_*i*_ (10^−12^ g d^-1^) × 365.
2. $$F_{DOC}=\Sigma_{i=1}^{12}Q_{i}\times C_{iDOC}$$
  Where *F*_*DOC*_ represents the annual flux of DOC (Tg yr^-1^), *Q*_*i*_ is the total water discharge (m^3^) in the _i_th month of a given year, and *C*_*iDOC*_ (mg L^-1^) is the monthly mean concentration of DOC for the _i_th month of a given year [30]. Specifically, *F*_*DOC*_ (Tg yr^-1^) = *C*_*i*_ (mg L^-1^) × *Q*_*i*_ (m^3^) × 12 = *C*_*i*_ (mg/1000 m^3^) × *Q*_*i*_ (m^3^) × 12 = *C*_*i*_ × *Q*_*i*_ (10^-12^g) × 12
3. Annual DOC export was calculated from the following formula:
  $$F_{DOC}=Q\times C$$
  Where *F*_*DOC*_ represents the annual flux of DOC (Tg yr^-1^), Q is the discharge (m^3^ yr^-1^) and C is the DOC concentration (mg L^-1^).

To correlate the spatial variability in riverine DOC against our set of drivers, the river network needed to be extracted by ArcGIS. First, the China DEM, at a 1-km spatial resolution, as provided by Cold and Arid Regions Sciences Data Center (http://westdc.westgis.ac.cn), was added to ArcGIS, and the Hydrology Modelling tool was plugged into ArcGIS at the same time. Second, based on the DEM of China in the ArcMap and after filling the sink DEM of China, calculating flow direction, flow Accumulation, Stream Net and Watershed, the range of river network basins in China was generated. Third, we extracted each field catchment from the collected and measured DOC sites based on the range of river network basins in China and then corresponded these data to certain statistics of the field catchment. In the same way, the contents of annual NPP from each regional catchment were extracted using ArcGIS.

A variety of environmental factors influences the DOC concentrations in the rivers. This regional scale study further highlights the importance of meteorological factors (temperature and precipitation), and vegetation productivity (NPP) in controlling DOC export from terrestrial and aquatic ecosystems. We used ArcGIS 9.3 to extract river networks and calculate the average monthly precipitation and temperature, annual average net primary productivity (NPP)in each regional catchment area. The statistical significance of differences in the correlation between the spatial variability of DOC concentrations and potential drivers (temperature, precipitation and NPP) was calculated a using linear regressive model, and the correlation between the discharge rate and DOC concentrations and flux was calculated using nonlinear regression.

## 3. Results

### 3.1 Total budget of mean annual DOC flux transported from the three rivers to the ocean

We estimated that the flux of DOC exported by the three Chinese rivers to the ocean was 2.73 Tg yr^-1^ (Table 2). Specifically, of the three rivers, the Yangtze River had the highest annual DOC flux (1.85 Tg), followed by the Pearl River (0.82 Tg) and the Yellow River (0.06 Tg).With regard to average DOC concentrations, the Yellow River had the highest value (2.70 mg L^-1^), followed by the Yangtze River (2.24 mg L^-1^) and the Pearl River (1.51 mg L^-1^) (Table 2).

**Table 2:** Concentrations and flux of DOC exported to the ocean (annual mean±SD) by three rivers in China.

River	Location	[DOC]	DOC flux
		mg L^-1^	Tg yr^-1^
Yangtze River	Datong station	2.24±0.53	1.85
Yellow River	Lijin station	2.70±0.38	0.06
Pearl River	Xjiang (Makou station)	1.40±0.51	0.32
	Beijiang (Hekou station)	1.52±0.64	0.23
	Dongjiang (Boluo station)	1.59±0.70	0.27

### 3.2 Correlation between discharge and DOC flux and concentrations

**Fig 2:**
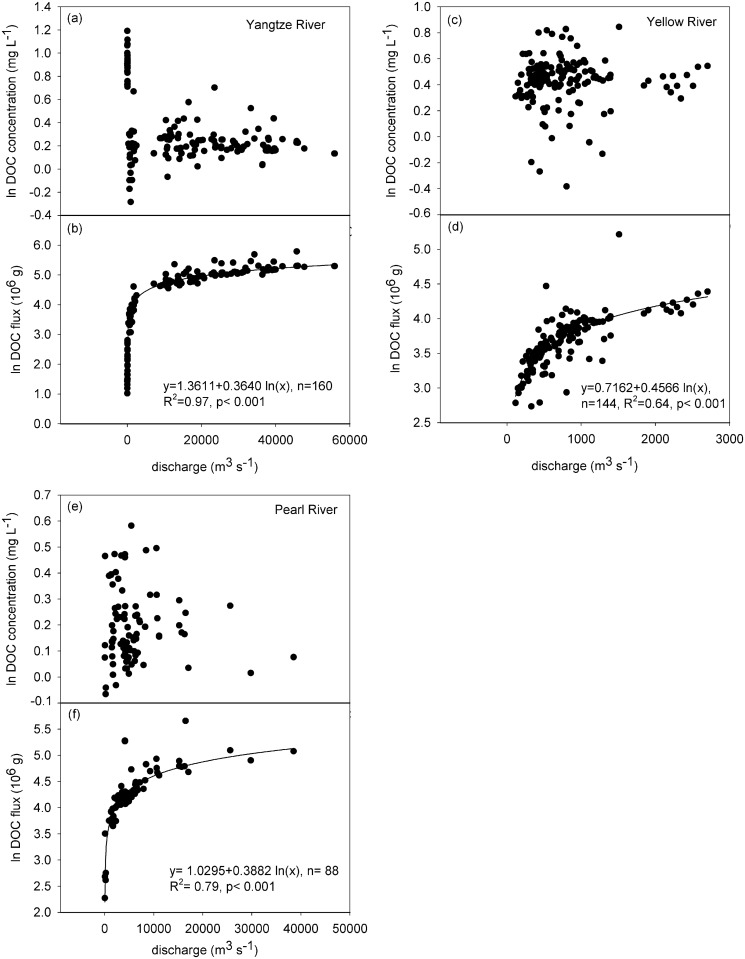
Correlation between the natural logarithm of DOC flux and concentrations and discharge from the three rivers.

The correlation between the discharge and DOC flux following increased discharge of DOC flux yields exponential growth in the three rivers (Fig 2b, 2d and 2f).Specifically, in the Yellow River (Fig 2d), which is located in an arid and semi-arid climate, the logarithm of DOC flux obviously exponentially increases followed increased discharge, compared with Yangtze River (Fig 2b) and Pearl River (Fig 2f). However, the logarithm of DOC concentrations did not follow increased discharge in the three rivers. For the Yellow River (Fig 2c), a high DOC concentrations mostly occurred during the spring flood period, in which the water discharge increased as a result of melting of ice and snow. In contrast, for the Yangtze River (Fig 2a) and Pearl River (Fig 2e), there were no large fluctuations in the logarithm of DOC concentrations based on increasing discharge.

### 3.3 Seasonal variation in DOC concentrations and flux

**Fig 3:**
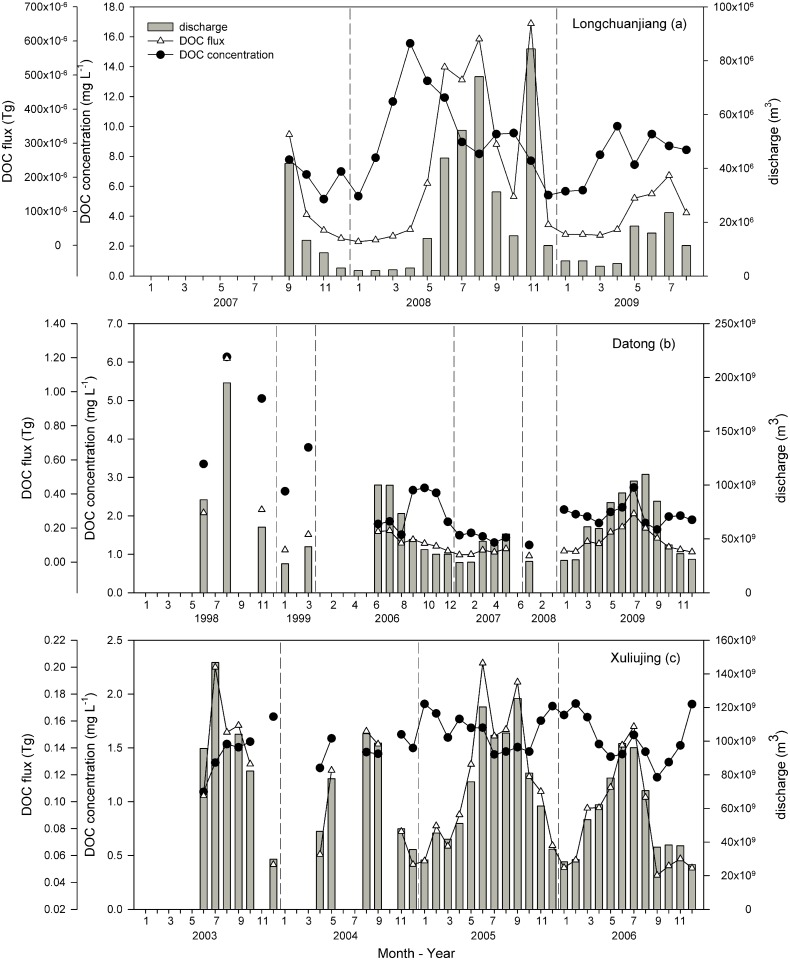
Seasonal and temporal variations of monthly discharge, DOC concentrations and flux from the mainstream and tributary hydrology stations along the Yangtze River. The dashed line represents the interval from year to year. (Date: mm–yy)

For the Yangtze River, the DOC flux was greatly influenced by the discharge rate while DOC concentrations displayed clear seasonal variation. Specifically, the highest DOC concentration (15.56 mg L^-1^) was detected in the Longchuanjiang (Fig 3a), a tributary of the Yangtze River, in April 2008. A flood occurred during autumn 2008, resulting in a higher water discharge in 2008 than was observed in 2007. While in the mainstream, the variation in DOC concentration achieved its highest value in the spring at the Datong station (Fig 3b). In addition, extreme events were important for the organic carbon cycling during a flood in August 1998. In the Xuliujing station, the variation of DOC concentration showed a similar pattern was seen for Datong, although the dynamic of DOC was delayed until the winter and subsequent spring (Fig 3c).

**Fig 4:**
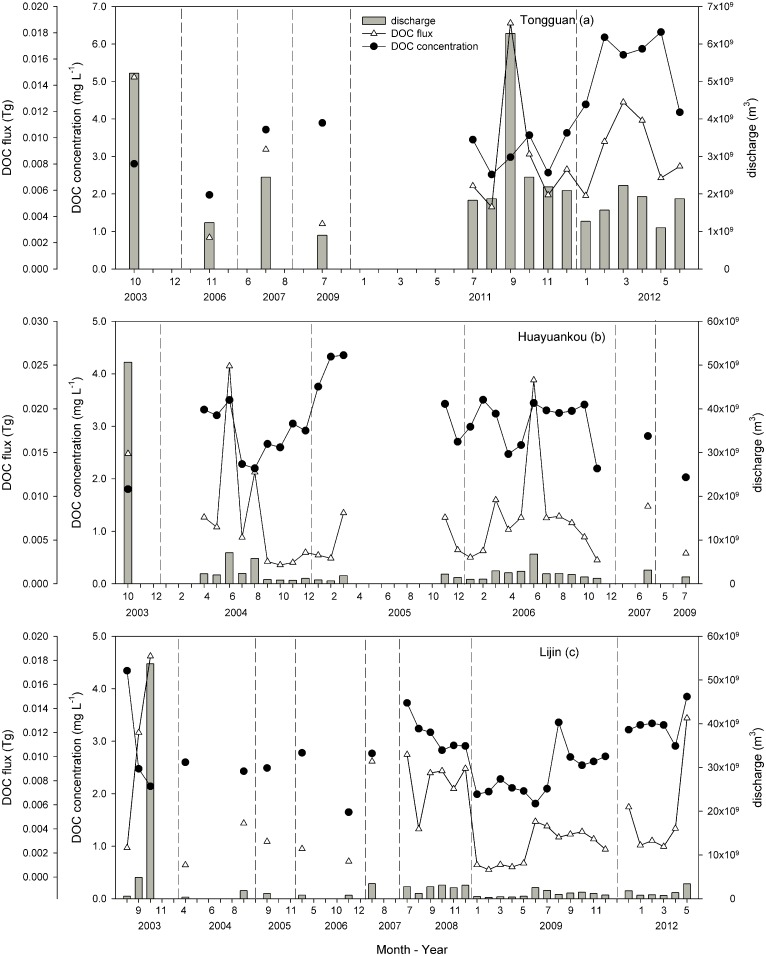
Seasonal and temporal variations of monthly discharge, DOC concentrations and flux along the mainstream of the Yellow River. The dashed line represents the interval from year to year. (Date: mm–yy)

Compared with the Yangtze, the Yellow River experiences intense soil erosion, increased turbidity and reduced discharge, and the DOC concentrations at the three Yellow River testing stations demonstrated seasonal change. At the Tongguan station, the DOC concentrations reached 6.18 mg L^-1^ in February 2012, which coincided with the melting period of ice and snow (Fig 4a). However, the DOC concentrations displayed more complex seasonal variations at the Huayuankou and Lijin stations (Fig 4b and 4c). In general, high DOC concentrations occurred during two periods. One period was from early February to March, which resulted from ice and snow melt; the other was the period when man-made flows were released downstream. In addition, at three stations, the discharge of DOC reached its highest levels following flooding in October 2003. Based on all of the sampling results, the highest mean DOC concentrations were detected at the Tongguan station, followed by the Toudaoguai, Huayuankou and Lijin stations (S1 Table).

**Fig 5:**
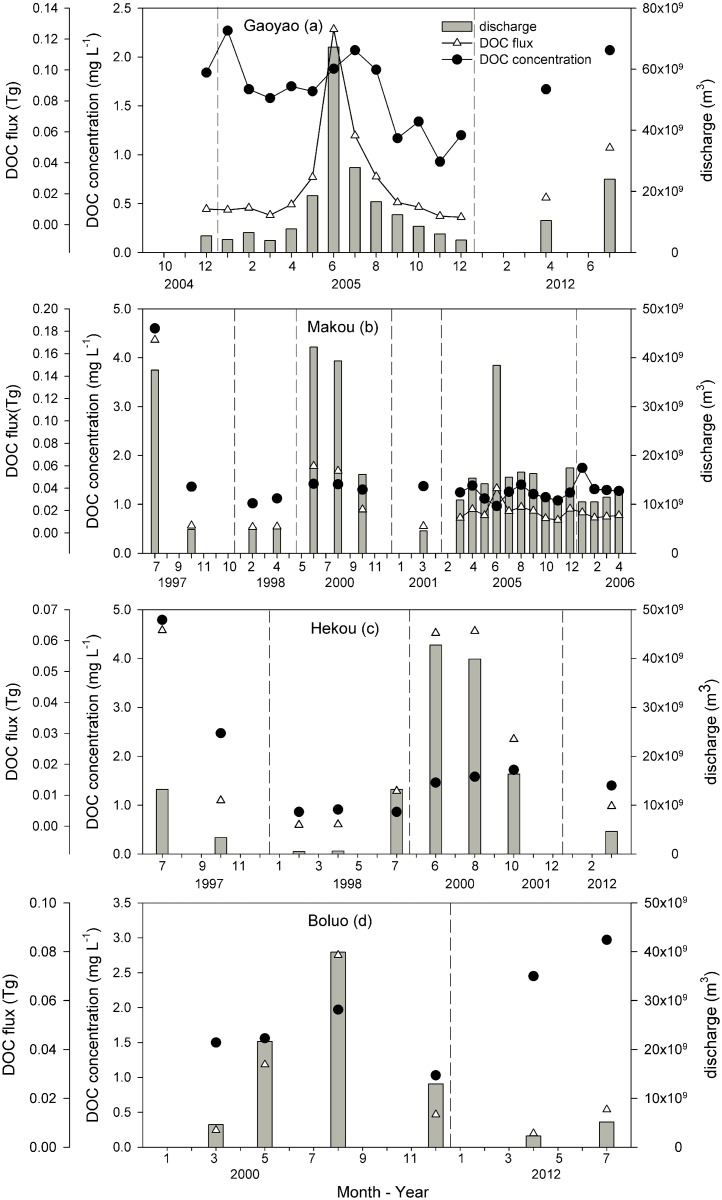
Seasonal and temporal variations of monthly discharge, DOC concentrations and flux from the main and tributary hydrology stations along the Pearl River. The dashed line represents the interval from year to year. (Date: mm–yy)

The Pearl River is composed of the Xijiang, Beijiang and Dongjiang tributaries. To compare the seasonal variation in DOC concentrations and flux, which were chosen to be representative of hydrological stations for the Gaoyao and Makou station (Xijiang), Hekou (Beijiang) and Boluo (Dongjiang). Specifically, there was a clear seasonal variation of DOC concentration at the Gaoyao station (Fig 5a). During an extreme flood in June 2005, although the riverine discharge reached its highest value, the DOC concentration did not reach its highest value, which explained the dilution effects. At the Makou station (Fig 5b), the DOC concentrations showed a relatively constant trend (excluding the 1997 flood, during which the DOC concentrations were as high as 4.60 mgL^-1^). At the Hekou and Boluo station (Fig 5c and 5d), there was no large fluctuation in DOC concentrations (except for a flood that occurred in July 1997 at the Hekou station).

### 3.4 Effects of meteorological factors and NPP on DOC concentrations and flux

#### 3.4.1 Effects of meteorological factors (temperature and precipitation)

**Fig 6:**
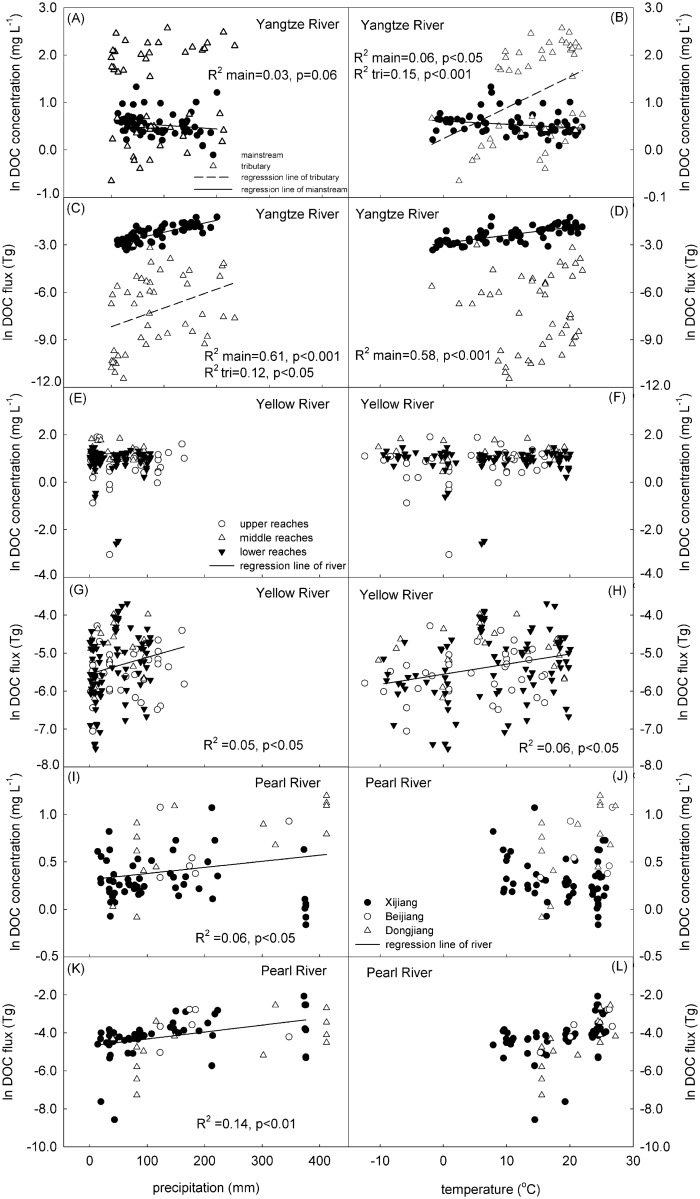
Variation of the natural logarithm of monthly DOC concentrations and flux on monthly temperature and precipitation in the Yangtze River, Yellow River and Pearl River catchments.

For both the mainstream and the tributary of the Yangtze River, we found a positive correlation between precipitation and DOC flux and concentrations (Fig 6C). However, only a weakly positive correlation between precipitation and DOC concentrations (Fig 6A) was detected for the mainstream alone. We also found that although there was a significantly positive correlation between temperature and DOC concentration (Fig 6B) for the Yangtze, a significantly positive correlation between temperature and DOC flux existed only in the mainstream (Fig 6D) and not in the tributary.

No significant relationships were detected between DOC concentrations and precipitation or temperature in the Yellow River (Fig 6E and 6F). There was, however, a weak positive correlation between DOC flux and both precipitation and temperature (Fig 6G and 6H). In addition, our results indicated that there was no significant correlation between temperature and DOC concentrations and flux in the Pearl River (Fig 6J and 6L). However, significantly positive correlations were determined between precipitation and both DOC concentrations and flux (Fig 6I and 6K) in the Pearl River.

#### 3.4.2 Effects of NPP

**Fig 7:**
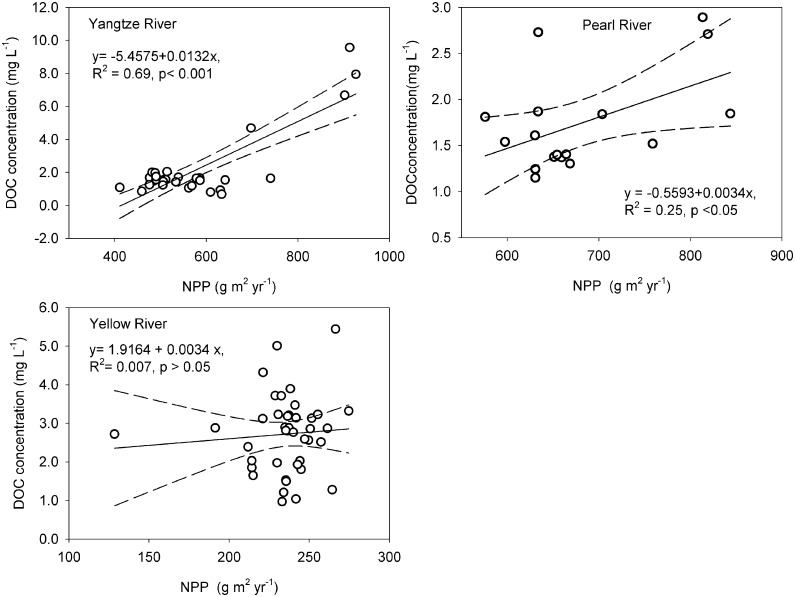
The relationship between DOC concentrations and annual average NPP in the Yangtze, Pearl and Yellow River catchments. The dotted lines represent the 95% confidence interval.

Our analysis of the NPP in regional catchments that corresponded to the DOC concentrations (Fig 7) revealed that the annual NPP had a significant positive correlation with the DOC concentrations in both the Yangtze and Pearl Rivers but not in the Yellow River. Specifically, for the Pearl River, a high annual NPP value was found in the Dongjiang tributary, and a low value NPP annual was found in the Xijiang tributary. In the Yangtze River datasets collected from the mainstream and tributary for this study, a high annual NPP value was found in the tributary. However, for the Yellow River, there was no positive relationship between the annual NPP and the riverine DOC concentration. There were no obvious regional changes to the annual NPP values of the Yellow river, which mainly ranged from 211 to 274 g m^2^ yr^-1^.

## 4. Discussion

### 4.1 Spatial and temporal patterns of DOC concentrations and flux

Most of the previous papers published on DOC in rivers were based on individual study that was performed in China [12,34]. We integrated the available DOC database to explore the spatial distribution of DOC transport patterns in different field sites from the three rivers. Compared with the flux of dissolved organic carbon in major world rivers (S3 Table). Such as, the flux of DOC in Mississippi river (budget of DOC flux is 3.5 x 10^12^ g/yr) [35] is larger than Yangtze River, Yellow river and Pearl River, respectively. The mean annual flux of DOC exported by the three rivers to the oceans accounted for approximately 1.6% of the global DOC flux [3]. Because of the various climates zone locations in the three river basins, there is a marked difference in the DOC concentrations and flux. Specifically, the DOC flux of the Yellow River was much lower than that of either the Yangtze or Pearl Rivers. The limiting factors for the DOC flux were low levels of precipitation, a high rate of evapotranspiration [36] and less vegetation coverage [37]. In addition, when comparing the DOC flux to that estimated in previous studies [12,31], it appeared that the DOC flux in the Pearl River that we estimated (see Table 3) is slightly higher than the historical values. This biased estimate was largely caused by the changes in the discharge rates during the different years that Tao et al. [11] carried out a 1-year analysis of riverine dissolved organic carbon (DOC) concentrations from the Xijiang tributary of Pearl River. However, in 2005, during their continuous sampling, there was a high water level month in the hydrological year. Taken together, these results underscore the major challenges in reliably estimating DOC flux, considering the limited number of samples available and the infrequency of annually measured DOC concentrations at some of the monitoring sites, particularly in the tributaries. In addition, estimates fail to account for the biogeochemical reactions that occur during the period of DOC transport.

**Table 3:** Comparison of flux of DOC exported to the ocean among the three Chinese rivers.

River	Year *	Number N*	Location	[DOC] mg L^-1^	DOC fluxTg yr^-1^	Source
**Yangtze River**	1998–2010	59	Datong	2.24	1.849	This study
	2008	2	Datong	1.77	1.465	Wang et al., 2010 [14]
	2006.06–2007.05	24	Datong	1.83	1.166	Zhang et al., 2014 [16]
	2009	12	Datong	2.03	1.58	Wang et al., 2012 [34]
**Yellow River**	2003–2012	226	Lijin	2.68	0.059	This study
	2009	12	Lijin	2.42	0.032	Wang et al., 2012 [34]
	1987	2	Lijin	5.30	0.20	Zhang et al., 1992 [38]
	2008.08–2012.07	192	Lijin	3.23	0.06	Ran et al., 2013 [39]
**Pearl River**	1997–2012	34	Pearl River*	1.51	0.82	This study
	2012	2	Pearl River	2.41	1.13	Zhang et al., 2013 [12]
	2000–2001	12	Pearl River	1.59	0.46	Wei et al., 2003 [31]

“Year” represent the period of measurement of DOC; “N” represent the measured the number of DOC during the measured period. Pearl River * includes the Xijiang (Makou station), Beijiang (Hekou station) and Dongjiang (Boluo station).

Transport dynamics of DOC have been analysed with respect to temporal variations. During the wet season, increased precipitation may lead to increased discharge, which lowers the DOC concentrations via dilution [40]. This was not consistent with our result for the variation in DOC concentration. Particularly in the Yellow River, the highest DOC concentration occurred in the spring, whereas discharge tended to be low during this period. Although the water discharge from the spring floods was significantly lower than that in the wet season, during the spring melting period, the DOC that was stored within the upper soil horizons was flushed out into the river, thus causing a sharp increase in DOC concentration [39]. The Yangtze River and the Pearl River had the same DOC variation patterns in the spring. When the mainstream was compared with the tributary of the Yangtze River, the tributary had a higher DOC concentration. Wu et al. [41] found that soil erosion was the main source of river organic matter in tributaries north of the Yangtze mainstream, whereas input from vascular plants was the main source in the south tributary. This demonstrates that terrestrial vegetation production contributes a large fraction of the organic carbon in the Yangtze River. Overall, tributary input and human activities may irregularly increase DOC concentrations. Although the impacts of various human activities can be elucidated in river basins that exhibit diverse land-usage patterns, they are difficult to quantify [42].

The discharge and DOC flux showed a significant exponential increase in our study, which illustrated that discharge is a key factor for controlling the flux of DOC in the rivers of China. Previous studies have shown that DOC flux is controlled primarily by the water discharge rate [43, 3], which was consistent with our results. The insignificant relationship between DOC concentrations and water discharge in the three rivers was potentially due to their location indifferent climate zone and the different land uses of the three river basins. The Yellow River, which is located in arid-semiarid climate zones, had low rainfall, high evapotranspiration and a frost period in the winter [39] and covered Quaternary loess deposits in the middle reaches [44], which result in the phenomenon that the DOC concentration did not follow increases with increasing water discharge. For the Yangtze River, urban and agriculture pollution was the major contributor to the variation in DOC concentration [16], and this impacted the actual relationship between water discharge and DOC concentration. Additionally, Tao et al. [11] found that the relationship of the riverine DOC concentrations with water discharge in Xijiang tributary of Pearl River is not significant, which is consistent with our result. Furthermore, Thurman E. M. [24] claimed that the increased DOC concentrations that they find in British rivers were not affected by river discharge, though river discharge can often explain variations in DOC export. In addition, in order to determine whether have another independent predictor variable in this relationship, we have reported the correlation (S1 Fig) between DOC concentration and DOC flux, in which the results show that although there is a positive relationship between DOC concentration and DOC flux in all of three rivers, compared to the correlation between discharge and DOC flux, such correlation is weaker. Finally, from our statistical analysis, we can better understand that the discharge is a better contributing factor to DOC flux than DOC concentration.

### 4.2 Key factors impacting DOC concentrations in the three river catchments

#### 4.2.1 Precipitation and temperature

Increasing temperature can affect DOC export in different ways, depending on whether it is accompanied by increased or decreased precipitation [45]. Based on our study, an increase in temperature results in only a partial increase in the DOC concentrations. These results are related to the regional catchment climate zone, vegetation and hydrology. Specifically, the Yellow River climate over the basin is primarily arid-semiarid with annual average temperature of 8–14°Cin most parts of the basin and an annual average precipitation of 476 mm [28]. In particular, precipitation is highly seasonally variable. In the wet season, the DOC concentration decreased rapidly to a low level, which is because most of the leachable DOC in the surface soil had already been released into the river with the spring floods [39]; thus, the variation in the DOC concentrations did not parallel the increase in precipitation and temperature. Compared with the Yellow River, the Yangtze River has a relatively higher temperature and precipitation than the Yellow River. For the tributary of the Yangtze River, a variation in DOC concentration resulted mainly from regional land use and anthropogenic activity [15] and was not a direct effect of precipitation and temperature. As for the Pearl River, which is located in a subtropical climate zone, precipitation and temperature are not the limiting factors for DOC concentrations. Our results demonstrate that the characteristics of riverine DOC concentrations mainly are mainly due to geomorphologic and hydrological processes and land-use within the drainage basin of the Pearl River [11].

In this study, primarily we focused on distinguishing the single influence of temperature or precipitation on DOC in rivers. As we know, the regional temperature and precipitation could both influence on DOC and discharge in rivers, so identify the relationship between regional temperature and precipitation and DOC is necessary. As for the interaction of regional precipitation and temperate on the change of riverine DOC. Our results (S4 Table) show that for the Yangtze River, correlation between temperature and precipitation and DOC flux in mainstream is important. There is a strong correlation between DOC flux and precipitation in tributary, while there is positive correlation between DOC concentration and temperature. But, for the Yellow river, the interaction of temperature and precipitation for DOC is existing. Similarly, for the Peal River, there is also correlation between interaction of temperature and precipitation and DOC flux.

In addition, the trend between seasonal variation and the environmental factors is generally consistent (S2–S4 Figs). Specifically, we can see that temperature, precipitation and discharge are consistent with seasonal variation, except for discharge in Tongguan station from the Yellow River, mainly because of human activities (such as water and sediment regulation). As for the DOC concentration, our results (S2–S4 Figs) show that higher DOC concentration in winter (from December to February) than other seasons, explained by the storage of soil organic carbon and ice and snow melt between winter and spring resulted in a low discharge and high DOC concentration [39].

#### 4.2.2 NPP

NPP is an important component of all terrestrial carbon budgets. The terrestrial export of DOC depends on the net primary production (NPP) of the terrestrial system, plant material degradation to DOC in soils[24] and hydrological flushing of this DOC to surface waters [25, 26]. Some studies have shown that ^14^C of recent NPP is an importance source of DOC [46, 47, 48, 49]; therefore, the correlation between the annual NPP and the DOC concentration should be considered. The NPP patterns vary spatially due to the regional environmental conditions, climatic factors, and vegetation types [50]. In China, although there have been some studies on the spatio-temporal patterns of NPP in the three river basins [51, 52, 53], few studies have examined the correlation analysis between regional catchments of annual NPP and riverine DOC concentrations. Based on our results, we showed a significant positive correlation between regional catchments of annual NPP and riverine DOC concentrations, except for the Yellow River.

Specifically, in Northeast, and North China, inter-annual variations of NPP were significantly correlated with precipitation [50]. The Yellow River is located in the arid and semi-arid climate zone in the north of China and covers Quaternary loess deposits mainly in the middle reaches [44]. The headwater region of the Yellow River is covered with an alpine meadow ecosystem, and the vast middle reaches are sparsely vegetated with grasses and shrubs [54]. It has been suggested that the DOC concentration value in the headwater of Yellow River is higher than that in middle reaches, as Rui et al. [52] showed that NPP is highest in the forest and mountain scrub regions and lowest in desert regions and that it changes from above 800 gm^-2^yr^-1^ to below 50 gm^-2^yr^-1^ in the Yellow River. Generally, a low NPP leads to low organic carbon content in soil, particularly in the Loess deposit zones. The effect of rainfall on NPP is significant in the desert steppe region, whereas the effect of temperature on NPP is significant in the alpine vegetation region and the Qinhai-Xizang Plateau. The relationship between NPP and temperature is negative in an area where NPP is positively correlated with rainfall, but it is positive in an area where NPP is negatively correlated with rainfall [52], which potentially results in an insignificantly correlation between NPP and DOC concentration in the Yellow River. In South China, the correlation between NPP and precipitation was statistically not significant [50]. Because the Yangtze River and Pearl River are located in temperate and subtropical climate zones and in a warm and humid climate, the more abundant vegetation coverage increases the production of NPP above that seen in the Yellow River. Moreover, the annual NPP of vegetation in the Yangtze River is 262 g m^-2^yr^-1^, as much as 1.5 times the total country means [53]. In our study, the value of annual NPP was greater in the tributary than in the mainstream, which is explained by the dilution effect. Vegetation in the Pearl River is mainly a subtropical evergreen broad-leaved forest, with NPP of approximately 1328.61 g m^-2^yr^-1^, as much as 4.12 times the total country means [51]. Moreover, the value of NPP in the Dongjiang tributary was not significantly increased, whereas the value of NPP in Xijiang tributary was not significantly decreased [51]. This is partly supported our results that the value of NPP in the Dongjiang tributary was higher than the Xijiang tributary.

## 5. Conclusions

The database presented here integrates data collected in previous studies on the DOC concentrations and fluxes in three large Chinese rivers. We estimated the DOC fluxes from the three rivers to the ocean to be 2.73Tg yr^-1^, with the Yellow River < Pearl River < Yangtze River. Based on a long-term investigation into the spatial and seasonal variation in DOC concentrations, the three rivers exhibit strong seasonal variability in their DOC concentrations. In the Yellow River, which is located in the arid and semi-arid climate zone, the regional temperature and precipitation are important factors for variations in DOC concentrations. In contrast, for the Yangtze River and Pearl River, which are located in temperate and subtropical climate zones, regional NPP is the main factor linking with variation of DOC concentration in rivers. In addition, the DOC flux was highly influenced by the rate of water discharge from all the three rivers. Studies based on geographic information systems analysing the regional catchment variability of DOC concentrations and flux are essential for gaining a better understanding of the whole-river DOC transport in these river basins.

## Supporting Information

S1 Table Concentrations and flux of DOC from different hydrology stations in China. (DOC)

S2 Table Description of the sites used in this analysis. (DOCX)

S3 Table Compared DOC flux of three rivers in China with the worldwide rivers. (DOCX)

S4 Table Correlation DOC flux/concentration to interaction of temperature and precipitation in regional catchment from three rivers. (DOCX)

S5 Table PRISMA flow diagram. (DOC)

S1 Fig Correlation between the natural logarithm of DOC flux and natural logarithm of DOC concentrations from the three rivers. (TIF)

S2 Fig Seasonal variation of meteorological factors on DOC from Yangtze River. (TIF)

S3 Fig Seasonal variation of meteorological factors on DOC from Yellow River. (TIF)

S4 Fig Seasonal variation of meteorological factors on DOC from Pearl River. (TIF)
