# Supplementary material for: The Spatial and Temporal Distribution of Dissolved Organic Carbon Exported from Three Chinese Rivers to the China Sea
Source: PLoS One. 2016 Oct 18;11(10):e0165039. doi: 10.1371/journal.pone.0165039 (PMC5068779; doi:10.1371/journal.pone.0165039)
Supplement: S1 Table — (DOC) [file pone.0165039.s001.doc]

S1 Table Concentrations and flux of DOC from different hydrology stations in China

| **River** | **Location** | **Start** | **End** | **Number of measurement** | **Average [DOC]** | **Standard deviation** | **Total annual of DOC flux** |
| --- | --- | --- | --- | --- | --- | --- | --- |
|  |  | year | year | N***** | mg L^-1^ | SD | Tg yr^-1^ |
| Yangtze River | Datong station | 1998 | 2010 | 59 | 2.24 | 0.53 | 1.8496 |
|  | Xuliujing station | 2003 | 2006 | 39 | 1.59 | 0.16 | 1.2537 |
|  | Xiaohekou station (Longchuanjiang) | 2007 | 2009 | 24 | 8.55 | 2.13 | 0.0021 |
| Yellow River | Toudaoguai station | 2011 | 2012 | 48 | 3.60 | 1.47 | 0.0644 |
|  | Tongguan station | 2003 | 2012 | 57 | 4.22 | 1.47 | 0.1081 |
|  | Huayuankou station | 2003 | 2009 | 74 | 3.00 | 0.56 | 0.0949 |
|  | Lijin station | 2003 | 2012 | 226 | 2.70 | 0.38 | 0.0588 |
| Pearl River | Luodingjiang station(Xijiang) | 2005 | 2005 | 9 | 1.81 | 0.96 | 0.0039 |
|  | Wuzhou station (Xijiang) | 2005 | 2006 | 14 | 1.30 | 0.22 | 0.2515 |
|  | Gaoyao station (Xijiang) | 2004 | 2012 | 15 | 1.64 | 0.37 | 0.3230 |
|  | Makou station (Xijiang) | 1997 | 2006 | 21 | 1.41 | 0.51 | 0.3210 |
|  | Hekou station (Beijiang) | 1997 | 2001 | 7 | 1.52 | 0.64 | 0.2294 |
|  | Boluo station (Dongjiang) | 2000 | 2012 | 6 | 1.59 | 0.70 | 0.2674 |

Note: “N” represents the measured number of times of DOC from the start year to the end year.
