# Supplementary material for: The Spatial and Temporal Distribution of Dissolved Organic Carbon Exported from Three Chinese Rivers to the China Sea
Source: PLoS One. 2016 Oct 18;11(10):e0165039. doi: 10.1371/journal.pone.0165039 (PMC5068779; doi:10.1371/journal.pone.0165039)
Supplement: S2 Table — (DOCX) [file pone.0165039.s002.docx]

S2 Table Description of the sites used in this analysis

(Note: N* represent measured the number of DOC in a certain time; [DOC]* represent DOC concentrations.)

| **ID** | **River** | **Site Name** | **Longitude (° )** | **Latitude (°)** | **Measure period** | **N*** | **[DOC]*** | **Reference** |
| --- | --- | --- | --- | --- | --- | --- | --- | --- |
| 1 | Yellow River | Dari | 100.082 | 33.692 | 2009.07 | 1 | 2.72 | Liu et al., 2010 [1] |
| 2 | Yellow River | Lajia | 102.094 | 33.94 | 2007.07 | 1 | 1.85 | Liu et al., 2010 [1] |
| 2 | Yellow River | Lajia | 102.094 | 33.94 | 2009.07 | 1 | 5.01 | Liu et al., 2010 [1] |
| 3 | Yellow River | Guide | 101.541 | 36.045 | 2007.07 | 1 | 0.97 | Liu et al., 2010 [1] |
| 3 | Yellow River | Guide | 101.541 | 36.045 | 2009.07 | 1 | 3.47 | Liu et al., 2010 [1] |
| 4 | Yellow River | Yinchuan | 106.572 | 38.648 | 2006.11 | 1 | 1.21 | Liu et al., 2010 [1] |
| 4 | Yellow River | Yinchuan | 106.572 | 38.648 | 2007.07 | 1 | 1.50 | Liu et al., 2010 [1] |
| 4 | Yellow River | Yinchuan | 106.572 | 38.648 | 2009.07 | 1 | 2.90 | Liu et al., 2010 [1] |
| 5 | Yellow River | Baotou | 109.843 | 40.583 | 2006.11 | 1 | 2.39 | Liu et al., 2010 [1] |
| 5 | Yellow River | Baotou | 109.843 | 40.583 | 2007.07 | 1 | 1.65 | Liu et al., 2010 [1] |
| 5 | Yellow River | Baotou | 109.843 | 40.583 | 2009.07 | 1 | 2.03 | Liu et al., 2010 [1] |
| 6 | Yellow River | Lanzhou (Xincheng) | 104.107 | 36.224 | 2003.10 | 1 | 1.60 | Su et al., 2005 [2] |
| 6 | Yellow River | Lanzhou (Baishiqiao) | 104.107 | 36.224 | 2003.10 | 1 | 1.91 | Su et al., 2005 [2] |
| 6 | Yellow River | Lanzhou | 104.107 | 36.224 | 2003.10 | 1 | 0.74 | Zhang et al., 2013 [3] |
| 6 | Yellow River | Lanzhou | 104.107 | 36.224 | 2006.11 | 1 | 2.21 | Zhang et al., 2013 [3] |
| 6 | Yellow River | Lanzhou | 104.107 | 36.224 | 2007.07 | 1 | 1.57 | Zhang et al., 2013 [3] |
| 6 | Yellow River | Lanzhou | 104.107 | 36.224 | 2009.07 | 1 | 2.56 | Zhang et al., 2013 [3] |
| 6 | Yellow River | Lanzhou | 104.107 | 36.224 | 2006.11 | 1 | 0.42 | Xu et al., 2008 [4] |
| 6 | Yellow River | Lanzhou | 104.107 | 36.224 | 2003.10 | 1 | 0.91 | Liu et al., 2010 [1] |
| 6 | Yellow River | Lanzhou | 104.107 | 36.224 | 2006.11 | 1 | 1.20 | Liu et al., 2010 [1] |
| 6 | Yellow River | Lanzhou | 104.107 | 36.224 | 2007.07 | 1 | 1.49 | Liu et al., 2010 [1] |
| 6 | Yellow River | Lanzhou | 104.107 | 36.224 | 2009.07 | 1 | 3.18 | Liu et al., 2010 [1] |
| 7 | Yellow River | Toudaoguai | 111.39 | 40.43 | 2011.07 | 4 | 2.93 | Ran et al., 2013 [5] |
| 7 | Yellow River | Toudaoguai | 111.39 | 40.43 | 2011.08 | 4 | 3.27 | Ran et al., 2013 [5] |
| 7 | Yellow River | Toudaoguai | 111.39 | 40.43 | 2011.09 | 4 | 2.74 | Ran et al., 2013 [5] |
| 7 | Yellow River | Toudaoguai | 111.39 | 40.43 | 2011.10 | 4 | 3.09 | Ran et al., 2013 [5] |
| 7 | Yellow River | Toudaoguai | 111.39 | 40.43 | 2011.11 | 4 | 2.74 | Ran et al., 2013 [5] |
| 7 | Yellow River | Toudaoguai | 111.39 | 40.43 | 2011.12 | 4 | 2.51 | Ran et al., 2013 [5] |
| 7 | Yellow River | Toudaoguai | 111.39 | 40.43 | 2012.01 | 4 | 2.99 | Ran et al., 2013 [5] |
| 7 | Yellow River | Toudaoguai | 111.39 | 40.43 | 2012.02 | 4 | 2.97 | Ran et al., 2013 [5] |
| 7 | Yellow River | Toudaoguai | 111.39 | 40.43 | 2012.03 | 4 | 6.73 | Ran et al., 2013 [5] |
| 7 | Yellow River | Toudaoguai | 111.39 | 40.43 | 2012.04 | 4 | 6.60 | Ran et al., 2013 [5] |
| 7 | Yellow River | Toudaoguai | 111.39 | 40.43 | 2012.05 | 4 | 2.72 | Ran et al., 2013 [5] |
| 7 | Yellow River | Toudaoguai | 111.39 | 40.43 | 2012.06 | 4 | 3.89 | Ran et al., 2013 [5] |
| 8 | Yellow River | Sanmenxia | 111.45 | 34.81 | 2003.10 | 1 | 3.21 | Su et al., 2005 [2] |
| 9 | Yellow River | Xiaolangdi | 112.15 | 35.25 | 2003.10 | 1 | 3.20 | Su et al., 2005 [2] |
| 10 | Yellow River | Tongguan | 110.396 | 34.584 | 2003.10 | 1 | 2.46 | Zhang et al., 2013 [3] |
| 10 | Yellow River | Tongguan | 110.396 | 34.584 | 2006.11 | 1 | 1.55 | Zhang et al., 2013 [3] |
| 10 | Yellow River | Tongguan | 110.396 | 34.584 | 2007.07 | 1 | 3.71 | Zhang et al., 2013 [3] |
| 10 | Yellow River | Tongguan | 110.396 | 34.584 | 2009.07 | 1 | 3.40 | Zhang et al., 2013 [3] |
| 10 | Yellow River | Tongguan | 110.396 | 34.584 | 2003.10 | 1 | 3.12 | Su et al., 2005 [2] |
| 10 | Yellow River | Tongguan | 110.396 | 34.584 | 2011.07 | 4 | 3.45 | Ran et al., 2013 [5] |
| 10 | Yellow River | Tongguan | 110.396 | 34.584 | 2011.08 | 4 | 2.52 | Ran et al., 2013 [5] |
| 10 | Yellow River | Tongguan | 110.396 | 34.584 | 2011.09 | 4 | 2.98 | Ran et al., 2013 [5] |
| 10 | Yellow River | Tongguan | 110.396 | 34.584 | 2011.10 | 4 | 3.57 | Ran et al., 2013 [5] |
| 10 | Yellow River | Tongguan | 110.396 | 34.584 | 2011.11 | 4 | 2.57 | Ran et al., 2013 [5] |
| 10 | Yellow River | Tongguan | 110.396 | 34.584 | 2011.12 | 4 | 3.63 | Ran et al., 2013 [5] |
| 10 | Yellow River | Tongguan | 110.396 | 34.584 | 2012.01 | 4 | 4.39 | Ran et al., 2013 [5] |
| 10 | Yellow River | Tongguan | 110.396 | 34.584 | 2012.02 | 4 | 6.18 | Ran et al., 2013 [5] |
| 10 | Yellow River | Tongguan | 110.396 | 34.584 | 2012.03 | 4 | 5.71 | Ran et al., 2013 [5] |
| 10 | Yellow River | Tongguan | 110.396 | 34.584 | 2012.04 | 4 | 5.87 | Ran et al., 2013 [5] |
| 10 | Yellow River | Tongguan | 110.396 | 34.584 | 2012.05 | 4 | 6.32 | Ran et al., 2013 [5] |
| 10 | Yellow River | Tongguan | 110.396 | 34.584 | 2012.06 | 4 | 4.18 | Ran et al., 2013 [5] |
| 10 | Yellow River | Tongguan | 110.396 | 34.584 | 2003.10 | 1 | 2.85 | Liu et al., 2010 [1] |
| 10 | Yellow River | Tongguan | 110.396 | 34.584 | 2006.11 | 1 | 2.40 | Liu et al., 2010 [1] |
| 10 | Yellow River | Tongguan | 110.396 | 34.584 | 2007.07 | 1 | 3.73 | Liu et al., 2010 [1] |
| 10 | Yellow River | Tongguan | 110.396 | 34.584 | 2009.07 | 1 | 4.39 | Liu et al., 2010 [1] |
| 11 | Yellow River | Huayuankou | 113.415 | 34.939 | 2003.10 | 1 | 2.76 | Su et al., 2005 [2] |
| 11 | Yellow River | Huayuankou | 113.415 | 34.939 | 2004.04 | 1 | 3.32 | Su et al., 2005 [2] |
| 11 | Yellow River | Huayuankou | 113.415 | 34.939 | 2004.05 | 1 | 3.21 | Su et al., 2005 [2] |
| 11 | Yellow River | Huayuankou | 113.415 | 34.939 | 2004.06 | 1 | 3.50 | Su et al., 2005 [2] |
| 11 | Yellow River | Huayuankou | 113.415 | 34.939 | 2004.07 | 1 | 2.28 | Su et al., 2005 [2] |
| 11 | Yellow River | Huayuankou | 113.415 | 34.939 | 2004.08 | 1 | 2.20 | Su et al., 2005 [2] |
| 11 | Yellow River | Huayuankou | 113.415 | 34.939 | 2004.09 | 1 | 2.66 | Su et al., 2005 [2] |
| 11 | Yellow River | Huayuankou | 113.415 | 34.939 | 2004.10 | 1 | 2.60 | Su et al., 2005 [2] |
| 11 | Yellow River | Huayuankou | 113.415 | 34.939 | 2004.11 | 1 | 3.05 | Su et al., 2005 [2] |
| 11 | Yellow River | Huayuankou | 113.415 | 34.939 | 2004.12 | 1 | 2.92 | Su et al., 2005 [2] |
| 11 | Yellow River | Huayuankou | 113.415 | 34.939 | 2005.01 | 1 | 3.76 | Su et al., 2005 [2] |
| 11 | Yellow River | Huayuankou | 113.415 | 34.939 | 2005.02 | 1 | 4.33 | Su et al., 2005 [2] |
| 11 | Yellow River | Huayuankou | 113.415 | 34.939 | 2005.03 | 1 | 4.35 | Su et al., 2005 [2] |
| 11 | Yellow River | Huayuankou | 113.415 | 34.939 | 2003.10 | 1 | 1.97 | Zhang et al., 2013 [3] |
| 11 | Yellow River | Huayuankou | 113.415 | 34.939 | 2006.11 | 1 | 2.95 | Zhang et al., 2013 [3] |
| 11 | Yellow River | Huayuankou | 113.415 | 34.939 | 2007.07 | 1 | 2.78 | Zhang et al., 2013 [3] |
| 11 | Yellow River | Huayuankou | 113.415 | 34.939 | 2009.07 | 1 | 2.81 | Zhang et al., 2013 [3] |
| 11 | Yellow River | Huayuankou | 113.415 | 34.939 | 2005.11 | 4 | 3.43 | Zhang et al., 2013 [3] |
| 11 | Yellow River | Huayuankou | 113.415 | 34.939 | 2005.12 | 4 | 2.71 | Zhang et al., 2013 [3] |
| 11 | Yellow River | Huayuankou | 113.415 | 34.939 | 2006.01 | 4 | 2.99 | Zhang et al., 2013 [3] |
| 11 | Yellow River | Huayuankou | 113.415 | 34.939 | 2006.02 | 4 | 3.51 | Zhang et al., 2013 [3] |
| 11 | Yellow River | Huayuankou | 113.415 | 34.939 | 2006.03 | 4 | 3.24 | Zhang et al., 2013 [3] |
| 11 | Yellow River | Huayuankou | 113.415 | 34.939 | 2006.04 | 4 | 2.47 | Zhang et al., 2013 [3] |
| 11 | Yellow River | Huayuankou | 113.415 | 34.939 | 2006.05 | 4 | 2.64 | Zhang et al., 2013 [3] |
| 11 | Yellow River | Huayuankou | 113.415 | 34.939 | 2006.06 | 4 | 3.44 | Zhang et al., 2013 [3] |
| 11 | Yellow River | Huayuankou | 113.415 | 34.939 | 2006.07 | 4 | 3.30 | Zhang et al., 2013 [3] |
| 11 | Yellow River | Huayuankou | 113.415 | 34.939 | 2006.08 | 4 | 3.25 | Zhang et al., 2013 [3] |
| 11 | Yellow River | Huayuankou | 113.415 | 34.939 | 2006.09 | 4 | 3.29 | Zhang et al., 2013 [3] |
| 11 | Yellow River | Huayuankou | 113.415 | 34.939 | 2006.10 | 4 | 3.41 | Zhang et al., 2013 [3] |
| 11 | Yellow River | Huayuankou | 113.415 | 34.939 | 2006.11 | 4 | 3.61 | Zhang et al., 2013 [3] |
| 11 | Yellow River | Huayuankou | 113.415 | 34.939 | 2006.11 | 1 | 0.54 | Xu et al., 2008 [4] |
| 11 | Yellow River | Huayuankou | 113.415 | 34.939 | 2003.10 | 1 | 2.41 | Liu et al., 2010 [1] |
| 11 | Yellow River | Huayuankou | 113.415 | 34.939 | 2006.11 | 1 | 1.68 | Liu et al., 2010 [1] |
| 11 | Yellow River | Huayuankou | 113.415 | 34.939 | 2007.07 | 1 | 2.85 | Liu et al., 2010 [1] |
| 11 | Yellow River | Huayuankou | 113.415 | 34.939 | 2009.07 | 1 | 1.25 | Liu et al., 2010 [1] |
| 12 | Yellow River | Lijin | 118.648 | 37.684 | 2003.10 | 1 | 3.09 | Su et al., 2005 [2] |
| 12 | Yellow River | Lijin | 118.648 | 37.684 | 2003.10 | 1 | 2.46 | Zhang et al., 2013 [3] |
| 12 | Yellow River | Lijin | 118.648 | 37.684 | 2006.11 | 1 | 2.11 | Zhang et al., 2013 [3] |
| 12 | Yellow River | Lijin | 118.648 | 37.684 | 2007.07 | 1 | 2.83 | Zhang et al., 2013 [3] |
| 12 | Yellow River | Lijin | 118.648 | 37.684 | 2009.07 | 1 | 1.83 | Zhang et al., 2013 [3] |
| 12 | Yellow River | Lijin | 118.648 | 37.684 | 2008.06 | 1 | 2.73 | Zhang et al., 2013 [3] |
| 12 | Yellow River | Lijin | 118.648 | 37.684 | 2008.07 | 1 | 2.92 | Zhang et al., 2013 [3] |
| 12 | Yellow River | Lijin | 118.648 | 37.684 | 2003.08 | 1 | 4.34 | Zhang et al., 2004 [6] |
| 12 | Yellow River | Lijin | 118.648 | 37.684 | 2003.09 | 1 | 2.48 | Zhang et al., 2004 [6] |
| 12 | Yellow River | Lijin | 118.648 | 37.684 | 2006.11 | 1 | 0.64 | Xu et al., 2008 [4] |
| 12 | Yellow River | Lijin | 118.648 | 37.684 | 2008（2009,2010,2011）.07 | 16 | 3.73 | Ran et al., 2013 [5] |
| 12 | Yellow River | Lijin | 118.648 | 37.684 | 2008（2009,2010,2011）.08 | 16 | 3.24 | Ran et al., 2013 [5] |
| 12 | Yellow River | Lijin | 118.648 | 37.684 | 2008（2009,2010,2011）.09 | 16 | 3.17 | Ran et al., 2013 [5] |
| 12 | Yellow River | Lijin | 118.648 | 37.684 | 2008（2009,2010,2011）.10 | 16 | 2.83 | Ran et al., 2013 [5] |
| 12 | Yellow River | Lijin | 118.648 | 37.684 | 2008（2009,2010,2011）.11 | 16 | 2.92 | Ran et al., 2013 [5] |
| 12 | Yellow River | Lijin | 118.648 | 37.684 | 2008（2009,2010,2011）.12 | 16 | 2.91 | Ran et al., 2013 [5] |
| 12 | Yellow River | Lijin | 118.648 | 37.684 | （2009,2010,2011）2012.01 | 16 | 3.22 | Ran et al., 2013 [5] |
| 12 | Yellow River | Lijin | 118.648 | 37.684 | （2009,2010,2011）2012.02 | 16 | 3.31 | Ran et al., 2013 [5] |
| 12 | Yellow River | Lijin | 118.648 | 37.684 | （2009,2010,2011）2012.03 | 16 | 3.34 | Ran et al., 2013 [5] |
| 12 | Yellow River | Lijin | 118.648 | 37.684 | （2009,2010,2011）2012.04 | 16 | 3.31 | Ran et al., 2013 [5] |
| 12 | Yellow River | Lijin | 118.648 | 37.684 | （2009,2010,2011）2012.05 | 16 | 2.91 | Ran et al., 2013 [5] |
| 12 | Yellow River | Lijin | 118.648 | 37.684 | （2009,2010,2011）2012.06 | 16 | 3.85 | Ran et al., 2013 [5] |
| 12 | Yellow River | Lijin | 118.648 | 37.684 | 2009.01 | 1 | 1.99 | Wang et al., 2012 [7] |
| 12 | Yellow River | Lijin | 118.648 | 37.684 | 2009.02 | 1 | 2.04 | Wang et al., 2012 [7] |
| 12 | Yellow River | Lijin | 118.648 | 37.684 | 2009.03 | 1 | 2.28 | Wang et al., 2012 [7] |
| 12 | Yellow River | Lijin | 118.648 | 37.684 | 2009.04 | 1 | 2.11 | Wang et al., 2012 [7] |
| 12 | Yellow River | Lijin | 118.648 | 37.684 | 2009.05 | 1 | 2.05 | Wang et al., 2012 [7] |
| 12 | Yellow River | Lijin | 118.648 | 37.684 | 2009.06 | 1 | 1.81 | Wang et al., 2012 [7] |
| 12 | Yellow River | Lijin | 118.648 | 37.684 | 2009.07 | 1 | 2.77 | Wang et al., 2012 [7] |
| 12 | Yellow River | Lijin | 118.648 | 37.684 | 2009.08 | 1 | 3.36 | Wang et al., 2012 [7] |
| 12 | Yellow River | Lijin | 118.648 | 37.684 | 2009.09 | 1 | 2.70 | Wang et al., 2012 [7] |
| 12 | Yellow River | Lijin | 118.648 | 37.684 | 2009.10 | 1 | 2.54 | Wang et al., 2012 [7] |
| 12 | Yellow River | Lijin | 118.648 | 37.684 | 2009.11 | 1 | 2.62 | Wang et al., 2012 [7] |
| 12 | Yellow River | Lijin | 118.648 | 37.684 | 2009.12 | 1 | 2.71 | Wang et al., 2012 [7] |
| 12 | Yellow River | Lijin | 118.648 | 37.684 | 2003.10 | 1 | 2.93 | Liu et al., 2010 [1] |
| 12 | Yellow River | Lijin | 118.648 | 37.684 | 2006.11 | 1 | 2.20 | Liu et al., 2010 [1] |
| 12 | Yellow River | Lijin | 118.648 | 37.684 | 2007.07 | 1 | 2.71 | Liu et al., 2010 [1] |
| 12 | Yellow River | Lijin | 118.648 | 37.684 | 2009.07 | 1 | 1.69 | Liu et al., 2010 [1] |
| 12 | Yellow River | Lijin | 118.648 | 37.684 | 2008.06-07 | 1 | 2.69 | Liu et al., 2010 [1] |
| 12 | Yellow River | Lijin | 118.648 | 37.684 | 2004.04 | 1 | 2.60 | Gu et al., 2009 [8] |
| 12 | Yellow River | Lijin | 118.648 | 37.684 | 2004.09 | 1 | 2.43 | Gu et al., 2009 [8] |
| 12 | Yellow River | Lijin | 118.648 | 37.684 | 2005.09 | 1 | 2.49 | Gu et al., 2009 [8] |
| 12 | Yellow River | Lijin | 118.648 | 37.684 | 2006.04 | 1 | 2.78 | Gu et al., 2009 [8] |
| 12 | Yellow River | Lijin | 118.648 | 37.684 | 2009.05 | 1 | 2.49 | Yin et al., 2010 [9] |
| 12 | Yellow River | Lijin | 118.648 | 37.684 | 2005.06-07 | 1 | 2.91 | Sun et al., 2007 [10] |
| 12 | Yellow River | Lijin | 118.648 | 37.684 | 2005.07 | 1 | 2.99 | Sun et al., 2007 [10] |
| 13 | Yellow River | Estuary | 118.517 | 37.9 | 2005.07 | 1 | 3.88 | Xia et al., 2011 [11] |
| 13 | Yellow River | Estuary | 118.517 | 37.9 | 2009.05 | 1 | 2.02 | Yin et al., 2010 [9] |
| 13 | Yellow River | Estuary | 118.517 | 37.9 | 2004.04; 2006.04 | 2 | 3.00 | Zhang et al., 2007 [12] |
| 13 | Yellow River | Estuary | 118.517 | 37.9 | 2004.09; 2005.09 | 2 | 2.60 | Zhang et al., 2007 [12] |
| 13 | Yellow River | Estuary | 118.517 | 37.9 | 1987（dry season） | 1 | 3.60 | Zhang et al., 1992 [13] |
| 13 | Yellow River | Estuary | 118.517 | 37.9 | 1987（wet season） | 1 | 7.0 | Zhang et al., 1992 [13] |
| 1 | Yangtze River | Yalongjiang | 101.615 | 26.980 | 2009.08-10 | 1 | 1.09 | Bao et al., 2012 [14] |
| 1 | Yangtze River | Yalongjiang | 101.615 | 26.980 | 2003.04-05 | 1 | 0.86 | Lin et al., 2007 [15] |
| 2 | Yangtze River | Longchuanjiang | 102.033 | 26.250 | 2007.09 | 1 | 7.78 | Lu et al., 2012 [16] |
| 2 | Yangtze River | Longchuanjiang | 102.033 | 26.250 | 2007.10 | 1 | 6.79 | Lu et al., 2012 [16] |
| 2 | Yangtze River | Longchuanjiang | 102.033 | 26.250 | 2007.11 | 1 | 5.14 | Lu et al., 2012 [16] |
| 2 | Yangtze River | Longchuanjiang | 102.033 | 26.250 | 2007.12 | 1 | 6.99 | Lu et al., 2012 [16] |
| 2 | Yangtze River | Longchuanjiang | 102.033 | 26.250 | 2008.01 | 1 | 5.34 | Lu et al., 2012 [16] |
| 2 | Yangtze River | Longchuanjiang | 102.033 | 26.250 | 2008.02 | 1 | 7.91 | Lu et al., 2012 [16] |
| 2 | Yangtze River | Longchuanjiang | 102.033 | 26.250 | 2008.03 | 1 | 11.67 | Lu et al., 2012 [16] |
| 2 | Yangtze River | Longchuanjiang | 102.033 | 26.250 | 2008.04 | 1 | 15.56 | Lu et al., 2012 [16] |
| 2 | Yangtze River | Longchuanjiang | 102.033 | 26.250 | 2008.05 | 1 | 13.05 | Lu et al., 2012 [16] |
| 2 | Yangtze River | Longchuanjiang | 102.033 | 26.250 | 2008.06 | 1 | 11.93 | Lu et al., 2012 [16] |
| 2 | Yangtze River | Longchuanjiang | 102.033 | 26.250 | 2008.07 | 1 | 8.97 | Lu et al., 2012 [16] |
| 2 | Yangtze River | Longchuanjiang | 102.033 | 26.250 | 2008.08 | 1 | 8.18 | Lu et al., 2012 [16] |
| 2 | Yangtze River | Longchuanjiang | 102.033 | 26.250 | 2008.09 | 1 | 9.49 | Lu et al., 2012 [16] |
| 2 | Yangtze River | Longchuanjiang | 102.033 | 26.250 | 2008.10 | 1 | 9.56 | Lu et al., 2012 [16] |
| 2 | Yangtze River | Longchuanjiang | 102.033 | 26.250 | 2008.11 | 1 | 7.71 | Lu et al., 2012 [16] |
| 2 | Yangtze River | Longchuanjiang | 102.033 | 26.250 | 2008.12 | 1 | 5.41 | Lu et al., 2012 [16] |
| 2 | Yangtze River | Longchuanjiang | 102.033 | 26.250 | 2009.01 | 1 | 5.67 | Lu et al., 2012 [16] |
| 2 | Yangtze River | Longchuanjiang | 102.033 | 26.250 | 2009.02 | 1 | 5.74 | Lu et al., 2012 [16] |
| 2 | Yangtze River | Longchuanjiang | 102.033 | 26.250 | 2009.03 | 1 | 8.11 | Lu et al., 2012 [16] |
| 2 | Yangtze River | Longchuanjiang | 102.033 | 26.250 | 2009.04 | 1 | 10.02 | Lu et al., 2012 [16] |
| 2 | Yangtze River | Longchuanjiang | 102.033 | 26.250 | 2009.05 | 1 | 7.45 | Lu et al., 2012 [16] |
| 2 | Yangtze River | Longchuanjiang | 102.033 | 26.250 | 2009.06 | 1 | 9.49 | Lu et al., 2012 [16] |
| 2 | Yangtze River | Longchuanjiang | 102.033 | 26.250 | 2009.07 | 1 | 8.70 | Lu et al., 2012 [16] |
| 2 | Yangtze River | Longchuanjiang | 102.033 | 26.250 | 2009.08 | 1 | 8.44 | Lu et al., 2012 [16] |
| 3 | Yangtze River | Niulanjiang | 103.203 | 27.058 | 2003.04-05 | 1 | 0.68 | Lin et al., 2007 [15] |
| 4 | Yangtze River | Minjiang | 104.163 | 29.038 | 2003.04-05 | 1 | 1.65 | Lin et al., 2007 [15] |
| 5 | Yangtze River | Tuojiang | 105.065 | 29.038 | 2003.04-05 | 1 | 4.69 | Lin et al., 2007 [15] |
| 6 | Yangtze River | Chishui | 105.653 | 28.548 | 2003.04-05 | 1 | 1.64 | Lin et al., 2007 [15] |
| 7 | Yangtze River | Jialingjiang | 106.182 | 30.312 | 1997.05 | 1 | 1.56 | Wang et al., 2010 [17] |
| 7 | Yangtze River | Jialingjiang | 106.182 | 30.312 | 2008.04 | 1 | 1.67 | Wang et al., 2010 [17] |
| 7 | Yangtze River | Jialingjiang | 106.182 | 30.312 | 2003.04-05 | 1 | 1.54 | Lin et al., 2007 [15] |
| 8 | Yangtze River | Wujiang | 107.652 | 29.391 | 1997.05 | 1 | 1.06 | Wang et al., 2010 [17] |
| 8 | Yangtze River | Wujiang | 107.652 | 29.391 | 2008.04 | 1 | 0.80 | Wang et al., 2010 [17] |
| 8 | Yangtze River | Wujiang | 107.652 | 29.391 | 2003.04-05 | 1 | 0.93 | Lin et al., 2007 [15] |
| 9 | Yangtze River | Hanjiang | 108.100 | 33.100 | 2003.04-05 | 1 | 2.01 | Lin et al., 2007 [15] |
| 9 | Yangtze River | Hanjiang | 108.100 | 33.100 | 2008.01 | 1 | 1.95 | Bao et al., 2012 [14] |
| 9 | Yangtze River | Hanjiang | 108.100 | 33.100 | 2009.08-10 | 1 | 1.33 | Bao et al., 2012 [14] |
| 9 | Yangtze River | Hanjiang | 108.100 | 33.100 | 2010.07-08 | 1 | 1.60 | Bao et al., 2012 [14] |
| 9 | Yangtze River | Hanjiang | 108.100 | 33.100 | 2007.11 | 1 | 1.25 | Tan et al., 2013 [18] |
| 9 | Yangtze River | Hanjiang | 108.100 | 33.100 | 2008.07 | 1 | 2.11 | Tan et al., 2013 [18] |
| 9 | Yangtze River | Hanjiang | 108.100 | 33.100 | 2008.11 | 1 | 1.08 | Tan et al., 2013 [18] |
| 9 | Yangtze River | Hanjiang | 108.100 | 33.100 | 2009.04 | 1 | 1.43 | Tan et al., 2013 [18] |
| 9 | Yangtze River | Hanjiang | 108.100 | 33.100 | 2009.08 | 1 | 2.14 | Tan et al., 2013 [18] |
| 9 | Yangtze River | Hanjiang | 108.100 | 33.100 | 2009.11 | 1 | 0.80 | Tan et al., 2013 [18] |
| 9 | Yangtze River | Hanjiang | 108.100 | 33.100 | 2010.01 | 1 | 0.52 | Tan et al., 2013 [18] |
| 10 | Yangtze River | Xiangjiang | 112.905 | 28.254 | 2003.04-05 | 1 | 1.19 | Lin et al., 2007 [15] |
| 11 | Yangtze River | Ganjiang | 115.707 | 28.274 | 2003.04-05 | 1 | 1.51 | Lin et al., 2007 [15] |
| 12 | Yangtze River | Datong | 117.183 | 30.767 | 1998.06 | 1 | 3.35 | Duan et al., 2008 [19] |
| 12 | Yangtze River | Datong | 117.183 | 30.767 | 1998.11 | 1 | 5.05 | Duan et al., 2008 [19] |
| 12 | Yangtze River | Datong | 117.183 | 30.767 | 1999.01 | 1 | 2.64 | Duan et al., 2008 [19] |
| 12 | Yangtze River | Datong | 117.183 | 30.767 | 1999.03 | 1 | 3.78 | Duan et al., 2008 [19] |
| 12 | Yangtze River | Datong | 117.183 | 30.767 | 2006.06 | 2 | 1.79 | Wang et al., 2010 [17] |
| 12 | Yangtze River | Datong | 117.183 | 30.767 | 2006.07 | 2 | 1.85 | Wang et al., 2010 [17] |
| 12 | Yangtze River | Datong | 117.183 | 30.767 | 2006.08 | 2 | 1.51 | Wang et al., 2010 [17] |
| 12 | Yangtze River | Datong | 117.183 | 30.767 | 2006.09 | 2 | 2.67 | Wang et al., 2010 [17] |
| 12 | Yangtze River | Datong | 117.183 | 30.767 | 2006.10 | 2 | 2.73 | Wang et al., 2010 [17] |
| 12 | Yangtze River | Datong | 117.183 | 30.767 | 2006.11 | 2 | 2.60 | Wang et al., 2010 [17] |
| 12 | Yangtze River | Datong | 117.183 | 30.767 | 2006.12 | 2 | 1.84 | Wang et al., 2010 [17] |
| 12 | Yangtze River | Datong | 117.183 | 30.767 | 2007.01 | 2 | 1.49 | Wang et al., 2010 [17] |
| 12 | Yangtze River | Datong | 117.183 | 30.767 | 2007.02 | 2 | 1.55 | Wang et al., 2010 [17] |
| 12 | Yangtze River | Datong | 117.183 | 30.767 | 2007.03 | 2 | 1.46 | Wang et al., 2010 [17] |
| 12 | Yangtze River | Datong | 117.183 | 30.767 | 2007.04 | 2 | 1.30 | Wang et al., 2010 [17] |
| 12 | Yangtze River | Datong | 117.183 | 30.767 | 2007.05 | 2 | 1.43 | Wang et al., 2010 [17] |
| 12 | Yangtze River | Datong | 117.183 | 30.767 | 2008.04 | 1 | 1.61 | Wang et al., 2010 [17] |
| 12 | Yangtze River | Datong | 117.183 | 30.767 | 2008.05 | 1 | 1.51 | Wang et al., 2010 [17] |
| 12 | Yangtze River | Datong | 117.183 | 30.767 | 2009.01 | 1 | 2.16 | Wang et al., 2012 [7] |
| 12 | Yangtze River | Datong | 117.183 | 30.767 | 2009.02 | 1 | 2.04 | Wang et al., 2012 [7] |
| 12 | Yangtze River | Datong | 117.183 | 30.767 | 2009.03 | 1 | 1.98 | Wang et al., 2012 [7] |
| 12 | Yangtze River | Datong | 117.183 | 30.767 | 2009.04 | 1 | 1.81 | Wang et al., 2012 [7] |
| 12 | Yangtze River | Datong | 117.183 | 30.767 | 2009.05 | 1 | 2.10 | Wang et al., 2012 [7] |
| 12 | Yangtze River | Datong | 117.183 | 30.767 | 2009.06 | 1 | 2.22 | Wang et al., 2012 [7] |
| 12 | Yangtze River | Datong | 117.183 | 30.767 | 2009.07 | 1 | 2.74 | Wang et al., 2012 [7] |
| 12 | Yangtze River | Datong | 117.183 | 30.767 | 2009.08 | 1 | 1.81 | Wang et al., 2012 [7] |
| 12 | Yangtze River | Datong | 117.183 | 30.767 | 2009.09 | 1 | 1.64 | Wang et al., 2012 [7] |
| 12 | Yangtze River | Datong | 117.183 | 30.767 | 2009.10 | 1 | 1.98 | Wang et al., 2012 [7] |
| 12 | Yangtze River | Datong | 117.183 | 30.767 | 2009.11 | 1 | 2.00 | Wang et al., 2012 [7] |
| 12 | Yangtze River | Datong | 117.183 | 30.767 | 2009.12 | 1 | 1.90 | Wang et al., 2012 [7] |
| 12 | Yangtze River | Datong | 117.183 | 30.767 | 2003.4-5 | 1 | 1.56 | Lin et al., 2007 [15] |
| 12 | Yangtze River | Datong | 117.183 | 30.767 | 2006.10-11 | 1 | 1.37 | Lin et al., 2007 [15] |
| 12 | Yangtze River | Datong | 117.183 | 30.767 | 2008.01 | 1 | 1.24 | Bao et al., 2012 [14] |
| 12 | Yangtze River | Datong | 117.183 | 30.767 | 2009.08-10 | 1 | 1.33 | Bao et al., 2012 [14] |
| 12 | Yangtze River | Datong | 117.183 | 30.767 | 2010.07-08 | 1 | 1.75 | Bao et al., 2012 [14] |
| 12 | Yangtze River | Datong | 117.183 | 30.767 | 2006.06 | 1 | 1.79 | Zhang et al., 2014 [20] |
| 12 | Yangtze River | Datong | 117.183 | 30.767 | 2006.07 | 1 | 1.85 | Zhang et al., 2014 [20] |
| 12 | Yangtze River | Datong | 117.183 | 30.767 | 2006.08 | 1 | 1.51 | Zhang et al., 2014 [20] |
| 12 | Yangtze River | Datong | 117.183 | 30.767 | 2006.09 | 1 | 2.67 | Zhang et al., 2014 [20] |
| 12 | Yangtze River | Datong | 117.183 | 30.767 | 2006.10 | 1 | 2.73 | Zhang et al., 2014 [20] |
| 12 | Yangtze River | Datong | 117.183 | 30.767 | 2006.11 | 1 | 2.60 | Zhang et al., 2014 [20] |
| 12 | Yangtze River | Datong | 117.183 | 30.767 | 2006.12 | 1 | 1.84 | Zhang et al., 2014 [20] |
| 12 | Yangtze River | Datong | 117.183 | 30.767 | 2007.01 | 1 | 1.49 | Zhang et al., 2014 [20] |
| 12 | Yangtze River | Datong | 117.183 | 30.767 | 2007.02 | 1 | 1.55 | Zhang et al., 2014 [20] |
| 12 | Yangtze River | Datong | 117.183 | 30.767 | 2007.03 | 1 | 1.46 | Zhang et al., 2014 [20] |
| 12 | Yangtze River | Datong | 117.183 | 30.767 | 2007.04 | 1 | 1.30 | Zhang et al., 2014 [20] |
| 12 | Yangtze River | Datong | 117.183 | 30.767 | 2007.05 | 1 | 1.43 | Zhang et al., 2014 [20] |
| 13 | Yangtze River | Xuliujing | 121.033 | 31.767 | 2003.06 | 1 | 1.08 | Lin et al., 2007 [15] |
| 13 | Yangtze River | Xuliujing | 121.033 | 31.767 | 2003.07 | 1 | 1.36 | Lin et al., 2007 [15] |
| 13 | Yangtze River | Xuliujing | 121.033 | 31.767 | 2003.08 | 1 | 1.54 | Lin et al., 2007 [15] |
| 13 | Yangtze River | Xuliujing | 121.033 | 31.767 | 2003.09 | 1 | 1.50 | Lin et al., 2007 [15] |
| 13 | Yangtze River | Xuliujing | 121.033 | 31.767 | 2003.10 | 1 | 1.55 | Lin et al., 2007 [15] |
| 13 | Yangtze River | Xuliujing | 121.033 | 31.767 | 2003.11 | 1 | 1.79 | Lin et al., 2007 [15] |
| 13 | Yangtze River | Xuliujing | 121.033 | 31.767 | 2004.01 | 1 | 1.91 | Lin et al., 2007 [15] |
| 13 | Yangtze River | Xuliujing | 121.033 | 31.767 | 2004.02 | 1 | 1.84 | Lin et al., 2007 [15] |
| 13 | Yangtze River | Xuliujing | 121.033 | 31.767 | 2004.03 | 1 | 1.51 | Lin et al., 2007 [15] |
| 13 | Yangtze River | Xuliujing | 121.033 | 31.767 | 2004.04 | 1 | 1.31 | Lin et al., 2007 [15] |
| 13 | Yangtze River | Xuliujing | 121.033 | 31.767 | 2004.05 | 1 | 1.59 | Lin et al., 2007 [15] |
| 13 | Yangtze River | Xuliujing | 121.033 | 31.767 | 2004.06 | 1 | 1.46 | Lin et al., 2007 [15] |
| 13 | Yangtze River | Xuliujing | 121.033 | 31.767 | 2004.07 | 1 | 1.45 | Lin et al., 2007 [15] |
| 13 | Yangtze River | Xuliujing | 121.033 | 31.767 | 2004.08 | 1 | 1.63 | Lin et al., 2007 [15] |
| 13 | Yangtze River | Xuliujing | 121.033 | 31.767 | 2004.09 | 1 | 1.50 | Lin et al., 2007 [15] |
| 13 | Yangtze River | Xuliujing | 121.033 | 31.767 | 2005.01 | 1 | 1.92 | Lin et al., 2007 [15] |
| 13 | Yangtze River | Xuliujing | 121.033 | 31.767 | 2005.02 | 1 | 1.79 | Lin et al., 2007 [15] |
| 13 | Yangtze River | Xuliujing | 121.033 | 31.767 | 2005.03 | 1 | 1.69 | Lin et al., 2007 [15] |
| 13 | Yangtze River | Xuliujing | 121.033 | 31.767 | 2005.04 | 1 | 1.77 | Lin et al., 2007 [15] |
| 13 | Yangtze River | Xuliujing | 121.033 | 31.767 | 2005.05 | 1 | 1.69 | Lin et al., 2007 [15] |
| 13 | Yangtze River | Xuliujing | 121.033 | 31.767 | 2005.06 | 1 | 1.69 | Lin et al., 2007 [15] |
| 13 | Yangtze River | Xuliujing | 121.033 | 31.767 | 2005.07 | 1 | 1.43 | Lin et al., 2007 [15] |
| 13 | Yangtze River | Xuliujing | 121.033 | 31.767 | 2005.08 | 1 | 1.47 | Lin et al., 2007 [15] |
| 13 | Yangtze River | Xuliujing | 121.033 | 31.767 | 2005.09 | 1 | 1.51 | Lin et al., 2007 [15] |
| 13 | Yangtze River | Xuliujing | 121.033 | 31.767 | 2005.10 | 1 | 1.47 | Lin et al., 2007 [15] |
| 13 | Yangtze River | Xuliujing | 121.033 | 31.767 | 2005.11 | 1 | 1.75 | Lin et al., 2007 [15] |
| 13 | Yangtze River | Xuliujing | 121.033 | 31.767 | 2005.12 | 1 | 1.89 | Lin et al., 2007 [15] |
| 13 | Yangtze River | Xuliujing | 121.033 | 31.767 | 2006.01 | 1 | 1.81 | Lin et al., 2007 [15] |
| 13 | Yangtze River | Xuliujing | 121.033 | 31.767 | 2006.02 | 1 | 1.91 | Lin et al., 2007 [15] |
| 13 | Yangtze River | Xuliujing | 121.033 | 31.767 | 2006.03 | 1 | 1.78 | Lin et al., 2007 [15] |
| 13 | Yangtze River | Xuliujing | 121.033 | 31.767 | 2006.04 | 1 | 1.53 | Lin et al., 2007 [15] |
| 13 | Yangtze River | Xuliujing | 121.033 | 31.767 | 2006.05 | 1 | 1.42 | Lin et al., 2007 [15] |
| 13 | Yangtze River | Xuliujing | 121.033 | 31.767 | 2006.06 | 1 | 1.44 | Lin et al., 2007 [15] |
| 13 | Yangtze River | Xuliujing | 121.033 | 31.767 | 2006.07 | 1 | 1.62 | Lin et al., 2007 [15] |
| 13 | Yangtze River | Xuliujing | 121.033 | 31.767 | 2006.08 | 1 | 1.46 | Lin et al., 2007 [15] |
| 13 | Yangtze River | Xuliujing | 121.033 | 31.767 | 2006.09 | 1 | 1.23 | Lin et al., 2007 [15] |
| 13 | Yangtze River | Xuliujing | 121.033 | 31.767 | 2006.10 | 1 | 1.37 | Lin et al., 2007 [15] |
| 13 | Yangtze River | Xuliujing | 121.033 | 31.767 | 2006.11 | 1 | 1.52 | Lin et al., 2007 [15] |
| 13 | Yangtze River | Xuliujing | 121.033 | 31.767 | 2006.12 | 1 | 1.91 | Lin et al., 2007 [15] |
| 14 | Yangtze River | Estuary | 121.650 | 32.650 | 2006.03-05 | 1 | 1.25 | Lin et al., 2007 [21] |
| 14 | Yangtze River | Estuary | 121.650 | 32.650 | 2003.06-08 | 1 | 1.74 | Lin et al., 2007 [21] |
| 14 | Yangtze River | Estuary | 121.650 | 32.650 | 2005.09-11 | 1 | 1.84 | Lin et al., 2007 [21] |
| 14 | Yangtze River | Estuary | 121.650 | 32.650 | 2005.12-2006.2 | 1 | 2.32 | Lin et al., 2007 [21] |
| 1 | Pearl River | Luodingjiang (Xijiang) | 111.529 | 22.711 | 2005.04-09 | 6 | 2.92 | Zhang et al., 2009 [22] |
| 1 | Pearl River | Luodingjiang (Xijiang) | 111.529 | 22.711 | 2005.10-12 | 1.5 | 1.32 | Zhang et al., 2009 [22] |
| 1 | Pearl River | Luodingjiang (Xijiang) | 111.529 | 22.711 | 2005.01-03 | 1.5 | 1.19 | Zhang et al., 2009 [22] |
| 2 | Pearl River | Gaoyao (Xijiang) | 112.467 | 23.050 | 2012.04; | 1 | 1.67 | Zhang et al., 2013 [23] |
| 2 | Pearl River | Gaoyao (Xijiang) | 112.467 | 23.050 | 2012.07 | 1 | 2.07 | Zhang et al., 2013 [23] |
| 2 | Pearl River | Gaoyao (Xijiang) | 112.467 | 23.050 | 2004.12 | 1 | 1.84 | Sun et al., 2006 [24] |
| 2 | Pearl River | Gaoyao (Xijiang) | 112.467 | 23.050 | 2005.01 | 1 | 2.27 | Sun et al., 2006 [24] |
| 2 | Pearl River | Gaoyao (Xijiang) | 112.467 | 23.050 | 2005.02 | 1 | 1.67 | Sun et al., 2006 [24] |
| 2 | Pearl River | Gaoyao (Xijiang) | 112.467 | 23.050 | 2005.03 | 1 | 1.58 | Sun et al., 2006 [24] |
| 2 | Pearl River | Gaoyao (Xijiang) | 112.467 | 23.050 | 2005.04 | 1 | 1.70 | Sun et al., 2006 [24] |
| 2 | Pearl River | Gaoyao (Xijiang) | 112.467 | 23.050 | 2005.05 | 1 | 1.65 | Sun et al., 2006 [24] |
| 2 | Pearl River | Gaoyao (Xijiang) | 112.467 | 23.050 | 2005.06 | 1 | 1.88 | Sun et al., 2006 [24] |
| 2 | Pearl River | Gaoyao (Xijiang) | 112.467 | 23.050 | 2005.07 | 1 | 2.07 | Sun et al., 2006 [24] |
| 2 | Pearl River | Gaoyao (Xijiang) | 112.467 | 23.050 | 2005.08 | 1 | 1.87 | Sun et al., 2006 [24] |
| 2 | Pearl River | Gaoyao (Xijiang) | 112.467 | 23.050 | 2005.09 | 1 | 1.17 | Sun et al., 2006 [24] |
| 2 | Pearl River | Gaoyao (Xijiang) | 112.467 | 23.050 | 2005.10 | 1 | 1.34 | Sun et al., 2006 [24] |
| 2 | Pearl River | Gaoyao (Xijiang) | 112.467 | 23.050 | 2005.11 | 1 | 0.93 | Sun et al., 2006 [24] |
| 2 | Pearl River | Gaoyao (Xijiang) | 112.467 | 23.050 | 2005.12 | 1 | 1.2 | Sun et al., 2006 [24] |
| 3 | Pearl River | Wuzhou (Xijiang) | 111.330 | 23.360 | 2005.03 | 1 | 1.29 | Tao et al., 2011 [25] |
| 3 | Pearl River | Wuzhou (Xijiang) | 111.330 | 23.360 | 2005.04 | 1 | 1.29 | Tao et al., 2011 [25] |
| 3 | Pearl River | Wuzhou (Xijiang) | 111.330 | 23.360 | 2005.05 | 1 | 1.24 | Tao et al., 2011 [25] |
| 3 | Pearl River | Wuzhou (Xijiang) | 111.330 | 23.360 | 2005.06 | 1 | 1.11 | Tao et al., 2011 [25] |
| 3 | Pearl River | Wuzhou (Xijiang) | 111.330 | 23.360 | 2005.07 | 1 | 1.15 | Tao et al., 2011 [25] |
| 3 | Pearl River | Wuzhou (Xijiang) | 111.330 | 23.360 | 2005.08 | 1 | 1.46 | Tao et al., 2011 [25] |
| 3 | Pearl River | Wuzhou (Xijiang) | 111.330 | 23.360 | 2005.09 | 1 | 1.27 | Tao et al., 2011 [25] |
| 3 | Pearl River | Wuzhou (Xijiang) | 111.330 | 23.360 | 2005.10 | 1 | 1.08 | Tao et al., 2011 [25] |
| 3 | Pearl River | Wuzhou (Xijiang) | 111.330 | 23.360 | 2005.11 | 1 | 1.36 | Tao et al., 2011 [25] |
| 3 | Pearl River | Wuzhou (Xijiang) | 111.330 | 23.360 | 2005.12 | 1 | 1.87 | Tao et al., 2011 [25] |
| 3 | Pearl River | Wuzhou (Xijiang) | 111.330 | 23.360 | 2006.01 | 1 | 1.38 | Tao et al., 2011 [25] |
| 3 | Pearl River | Wuzhou (Xijiang) | 111.330 | 23.360 | 2006.02 | 1 | 1.20 | Tao et al., 2011 [25] |
| 3 | Pearl River | Wuzhou (Xijiang) | 111.330 | 23.360 | 2006.03 | 1 | 1.44 | Tao et al., 2011 [25] |
| 3 | Pearl River | Wuzhou (Xijiang) | 111.330 | 23.360 | 2006.04 | 1 | 1.19 | Tao et al., 2011 [25] |
| 4 | Pearl River | Makou (Xijiang) | 112.840 | 23.030 | 1997.10 | 1 | 1.36 | Gao et al., 2002 [26] |
| 4 | Pearl River | Makou (Xijiang) | 112.840 | 23.030 | 1998.02 | 1 | 1.02 | Gao et al., 2002 [26] |
| 4 | Pearl River | Makou (Xijiang) | 112.840 | 23.030 | 1998.04 | 1 | 1.12 | Gao et al., 2002 [26] |
| 4 | Pearl River | Makou (Xijiang) | 112.840 | 23.030 | 2005.03 | 1 | 1.24 | Tao et al., 2011 [25] |
| 4 | Pearl River | Makou (Xijiang) | 112.840 | 23.030 | 2005.04 | 1 | 1.38 | Tao et al., 2011 [25] |
| 4 | Pearl River | Makou (Xijiang) | 112.840 | 23.030 | 2005.05 | 1 | 1.12 | Tao et al., 2011 [25] |
| 4 | Pearl River | Makou (Xijiang) | 112.840 | 23.030 | 2005.06 | 1 | 1.04 | Tao et al., 2011 [25] |
| 4 | Pearl River | Makou (Xijiang) | 112.840 | 23.030 | 2005.07 | 1 | 1.26 | Tao et al., 2011 [25] |
| 4 | Pearl River | Makou (Xijiang) | 112.840 | 23.030 | 2005.08 | 1 | 1.40 | Tao et al., 2011 [25] |
| 4 | Pearl River | Makou (Xijiang) | 112.840 | 23.030 | 2005.09 | 1 | 1.21 | Tao et al., 2011 [25] |
| 4 | Pearl River | Makou (Xijiang) | 112.840 | 23.030 | 2005.10 | 1 | 1.14 | Tao et al., 2011 [25] |
| 4 | Pearl River | Makou (Xijiang) | 112.840 | 23.030 | 2005.11 | 1 | 1.08 | Tao et al., 2011 [25] |
| 4 | Pearl River | Makou (Xijiang) | 112.840 | 23.030 | 2005.12 | 1 | 1.24 | Tao et al., 2011 [25] |
| 4 | Pearl River | Makou (Xijiang) | 112.840 | 23.030 | 2006.01 | 1 | 1.75 | Tao et al., 2011 [25] |
| 4 | Pearl River | Makou (Xijiang) | 112.840 | 23.030 | 2006.02 | 1 | 1.31 | Tao et al., 2011 [25] |
| 4 | Pearl River | Makou (Xijiang) | 112.840 | 23.030 | 2006.03 | 1 | 1.29 | Tao et al., 2011 [25] |
| 4 | Pearl River | Makou (Xijiang) | 112.840 | 23.030 | 2006.04 | 1 | 1.27 | Tao et al., 2011 [25] |
| 4 | Pearl River | Makou (Xijiang) | 112.840 | 23.030 | 2000.06 | 1 | 1.42 | Wei et al., 2003 [27] |
| 4 | Pearl River | Makou (Xijiang) | 112.840 | 23.030 | 2000.08 | 1 | 1.41 | Wei et al., 2003 [27] |
| 4 | Pearl River | Makou (Xijiang) | 112.840 | 23.030 | 2000.10 | 1 | 1.30 | Wei et al., 2003 [27] |
| 4 | Pearl River | Makou (Xijiang) | 112.840 | 23.030 | 2001.03 | 1 | 1.37 | Wei et al., 2003 [27] |
| 5 | Pearl River | Shijiao (Beijiang) | 112.960 | 23.550 | 2012.04 | 1 | 2.53 | Zhang et al., 2013 [23] |
| 5 | Pearl River | Shijiao (Beijiang) | 112.960 | 23.550 | 2010.07 | 1 | 2.93 | Zhang et al., 2013 [23] |
| 6 | Pearl River | Hekou (Beijiang) | 112.837 | 23.285 | 2000.06 | 1 | 1.46 | Wei et al., 2003 [27] |
| 6 | Pearl River | Hekou (Beijiang) | 112.837 | 23.285 | 2000.08 | 1 | 1.58 | Wei et al., 2003 [27] |
| 6 | Pearl River | Hekou (Beijiang) | 112.837 | 23.285 | 2000.10 | 1 | 1.72 | Wei et al., 2003 [27] |
| 6 | Pearl River | Hekou (Beijiang) | 112.837 | 23.285 | 2001.03 | 1 | 1.40 | Wei et al., 2003 [27] |
| 6 | Pearl River | Hekou (Beijiang) | 112.837 | 23.285 | 1997.10 | 1 | 2.47 | Gao et al., 2001 [28] |
| 6 | Pearl River | Hekou (Beijiang) | 112.837 | 23.285 | 1998.02 | 1 | 0.86 | Gao et al., 2001 [28] |
| 6 | Pearl River | Hekou (Beijiang) | 112.837 | 23.285 | 1998.04 | 1 | 0.91 | Gao et al., 2001 [28] |
| 7 | Pearl River | Boluo (Dongjiang) | 114.250 | 23.050 | 2012.04 | 1 | 2.45 | Zhang et al., 2013 [23] |
| 7 | Pearl River | Boluo (Dongjiang) | 114.250 | 23.050 | 2010.07 | 1 | 2.97 | Zhang et al., 2013 [23] |
| 7 | Pearl River | Boluo (Dongjiang) | 114.250 | 23.050 | 2000.05 | 1 | 1.56 | Wei et al., 2003 [27] |
| 7 | Pearl River | Boluo (Dongjiang) | 114.250 | 23.050 | 2000.08 | 1 | 1.97 | Wei et al., 2003 [27] |
| 7 | Pearl River | Boluo (Dongjiang) | 114.250 | 23.050 | 2000.11; | 1 | 1.03 | Wei et al., 2003 [27] |
| 7 | Pearl River | Boluo (Dongjiang) | 114.250 | 23.050 | 2001.03 | 1 | 1.50 | Wei et al., 2003 [27] |
| 8 | Pearl River | Humen (Dongjiang) | 113.220 | 23.110 | 2009.11 | 1 | 2.48 | Fu et al., 2014 [29] |
| 8 | Pearl River | Humen (Dongjiang) | 113.220 | 23.110 | 2010.06 | 1 | 3.07 | Fu et al., 2014 [29] |
| 9 | Pearl River | Estuary | 113.713 | 22.366 | 1996.07 | 1 | 3.13 | Dai et al., 2000 [30] |
| 9 | Pearl River | Estuary | 113.713 | 22.366 | 2004.02 | 1 | 2.96 | Lin et al., 2007 [21] |
| 9 | Pearl River | Estuary | 113.713 | 22.366 | 2005.08 | 1 | 1.76 | Lin et al., 2007 [21] |
| 9 | Pearl River | Estuary | 113.713 | 22.366 | 2006.03 | 1 | 2.88 | Lin et al., 2007 [21] |
| 9 | Pearl River | Estuary | 113.713 | 22.366 | 2005.03 | 1 | 2.15 | Ni et al., 2008 [31] |
| 9 | Pearl River | Estuary | 113.713 | 22.366 | 2005.04 | 1 | 1.73 | Ni et al., 2008 [31] |
| 9 | Pearl River | Estuary | 113.713 | 22.366 | 2005.05 | 1 | 1.48 | Ni et al., 2008 [31] |
| 9 | Pearl River | Estuary | 113.713 | 22.366 | 2005.06 | 1 | 1.19 | Ni et al., 2008 [31] |
| 9 | Pearl River | Estuary | 113.713 | 22.366 | 2005.07 | 1 | 1.08 | Ni et al., 2008 [31] |
| 9 | Pearl River | Estuary | 113.713 | 22.366 | 2005.08 | 1 | 1.68 | Ni et al., 2008 [31] |
| 9 | Pearl River | Estuary | 113.713 | 22.366 | 2005.09 | 1 | 1.63 | Ni et al., 2008 [31] |
| 9 | Pearl River | Estuary | 113.713 | 22.366 | 2005.10 | 1 | 1.55 | Ni et al., 2008 [31] |
| 9 | Pearl River | Estuary | 113.713 | 22.366 | 2005.11 | 1 | 1.70 | Ni et al., 2008 [31] |
| 9 | Pearl River | Estuary | 113.713 | 22.366 | 2005.12 | 1 | 2.39 | Ni et al., 2008 [31] |
| 9 | Pearl River | Estuary | 113.713 | 22.366 | 2006.01 | 1 | 1.86 | Ni et al., 2008 [31] |
| 9 | Pearl River | Estuary | 113.713 | 22.366 | 2006.02 | 1 | 1.75 | Ni et al., 2008 [31] |
| 9 | Pearl River | Estuary | 113.713 | 22.366 | 2007.04 | 1 | 3.82 | He et al., 2010 [32] |
| 9 | Pearl River | Estuary | 113.713 | 22.366 | 2001.05 | 1 | 1.43 | Callahan et al., 2004 [33] |
| 9 | Pearl River | Estuary | 113.713 | 22.366 | 2002.11 | 1 | 1.44 | Callahan et al., 2004 [33] |

**Cited References:**

1. Liu DM, Zhang LJ.Temporal and Spatial Distributions of Organic Carbon in the Huanghe (Yellow) River. Periodical of ocean university of China. 2010; 40(12), 105- 110. (In Chinese)

2. Su Z, Zhang LJ.Temporal and Spatial Distributions of Organic Carbon in the Huanghe (Yellow) River. Master thesis. Ocean University of China.2005. (In Chinese)

3. Zhang LJ, Wang L, Cai WJ, Liu DM, Yu ZG. Impact of human activities on organic carbon transport in the Yellow River. Biogeosciences.2013; 10, 2513-2524.

4. Xu X, Zhang LJ. Study of the carbon content and transportation character of The Yellow River in autumn. Master thesis.Ocean University of China. 2008. (In Chinese)

5. Ran L, Lu XX, Sun H, Han J, Li R, Zhang J.Spatial and seasonal variability of organic carbon transport in the Yellow River, China. Journal of Hydrology.2013; 498, 76-88.

6. Zhang X, Zhang LJ. Temporal and spatial distribution of organic carbon and its influence aspects in Huanghe estuary. Master thesis.Ocean University of China. 2004. (In Chinese)

7. Wang Xucheng, Ma H, Li R, Song Z, Wu J.Seasonal fluxes and source variation of organic carbon transported by two major Chinese Rivers: The Yellow River and Changjiang (Yangtze) River. Global Biogeochemical Cycles.2012; 26, GB2025, doi:10.1029/2011GB004130.

8. Gu D, Zhang L, Jiang L.The effects of estuarine processes on the fluxes of inorganic and organic carbon in the Yellow River estuary. Journal of Ocean University of China.2009; 8, 352-358.

9. Yin Peng, Zhang LJ. The study of carbon system Parameters and nutrients in theYe11ow River Estuary and adjacent waters. Master thesis. Ocean University of China. 2010. (In Chinese)

10. Sun Chao, Zhang LJ.Distribution and the Flux of Carbon in Different Periods of Yellow River. Ocean University of China. Master thesis. 2007. (In Chinese)

11. Xia B, Zhang L. Carbon distribution and fluxes of 16 rivers discharging into the Bohai Sea in summer. Acta Oceanologica Sinica.2011; 30, 43-54.

12. Zhang LJ, Zhang X, WangX, et al. Spatial and temporal distribution of particulate and dissolved organic carbon in Yellow River estuary. Advances in water science. 2007; 18(5), 674-682.

13. Zhang S, Wei-Bin G, Venugopalan I. Organic matter in large turbid rivers: the Huanghe and its estuary. Marine Chemistry.1992; 38, 53-68.

14. Bao H, Wu Ying.The sources, transportations and transformations of dissolved and particulate terrestrial organic matter in typical river and estuary systems. Doctoral Thesis. East China Normal University. 2012. (In Chinese)

15. Lin J, Dai M.The behavior and fluxof dissolved organic carbon in the Yangtze and Pearl riverestuary. Master thesis. Xiamen University. 2007. (In Chinese)

16. Lu XX, Li S, HeM, Zhou Y, Li L, Ziegler AD. Organic carbon fluxes from the upper Yangtze basin: an example of the Longchuanjiang River, China. Hydrological Processes.2012; 26, 1604-1616.

17. Wang M, Zhang LJ. Spatial and Temporal Transport of Carbon in Changjiang Mainstream and influences of Three Gorges Project. Master thesis.Ocean University of China. 2010. (In Chinese)

18. Tan X, Xia X, Zhao Q, Zhang Q. Temporal variations of benthic diatom community and its main influencing factors in a subtropical river, China. Environ Sci Pollut Res. 2013. DOI 10.1007/s11356-013-1898-0.

19. DuanS, Liang T, Zhang S, Wang L, Zhang X, Chen X. Seasonal changes in nitrogen and phosphorus transport in the lower Changjiang River before the construction of the Three Gorges Dam. Estuarine, Coastal and Shelf Science.2008; 79, 239-250.

20. Zhang LJ, Xue M, Wang M, Cai WJ, Wang L, Yu ZG. The spatiotemporal distribution of dissolved inorganic and organic carbon in the main stem of the Changjiang (Yangtze) River and the effect of the Three Gorges Reservoir. Journal of Geophysical Research-Biogeosciences.2014; 119, 741-757.

21. Lin J, Wu Y. Distributions of Dissolved organic Carbon and Particulate Organic Carbon in the Changjiang Estuary and its Adjacent Area. Master thesis. East China Normal University. 2007. (In Chinese)

22. Zhang S, Lu XX, Sun H, Han J, Higgitt DL. Geochemical characteristics and fluxes of organic carbon in a human-disturbed mountainous river (the Luodingjiang River) of the Zhujiang (Pearl River), China. Sci Total Environ.2009; 407, 815-25.

23. Zhang LK, Qin XQ, Yang H, Huang QB, Liu PY.Transported fluxes of the riverine carbon and seasonal variation in Pearl River basin. Environmental science. 2013; 34, 3025-3034. (In Chinese)

24. Sun H, Han J, Zhang S, Lu X. The impacts of ‘05.6’ extreme flood event on riverine carbon fluxes in Xijiang River. Chinese Science Bulletin. 2006; 51(23), 2773-2779. (In Chinese)

25. Tao Z, Gao QZ, GuoWP, Wang ZG, Zhang YL, Xie CJ, et al. Temporal and spatial variability of dissolved organic carbon concentration in the Xijiang River, South China. Journal of Mountain Science.2011; 8, 694-703.

26. Gao Q, Tao Z, Shen C, Sun Y,Yi W, Xing C. Riverine organic carbon in the Xijiang River (South China): seasonal variation in content and flux budget. Environmental Geology.2002; 41, 826-832.

27. Wei Xiuguo, Shen C. Study on riverine carbon flux and erosion of Zhujiang (Pearl ) river drainage basin(Ph.D. Dissertation). Chinese Academy of science.2003. (In Chinese)

28. Gao Q, Shen C, Sun Y, Yi W. A preliminary study on the organic carbon weathering fluxes in Beijiang river drainage. Environmental science. 2001; 22(2), 12-18.

29. Fu YC, Tang CG, Li J, Zhao YL, Zhong W, Zeng XT. Sources and transport of organic carbon from the Dongjiang River to the Humen outlet of the Pearl River, southern Chi. Journal of Geographical Sciences.2014; 24, 143-158.

30. DaiM, Martin J-M, Hua-sheng H, Zu-lin Z.Preliminary study on the dissolved and colloidal organic carbon in the Zhujiang River estuary. Chinese Journal of Oceanology and Limnology.2000; 18, 265-273.

31. Ni HG, Lu FH, Luo XL, Tian HY, Zeng EY. Riverine inputs of total organic carbon and suspended particulate matter from the Pearl River Delta to the coastal ocean off South China. Mar Pollut Bull.2008; 56, 1150-7.

32. He B, Dai M, Zhai W, Wang L, Wang K, Chen J, et al. Distribution, degradation and dynamics of dissolved organic carbon and its major compound classes in the Pearl River estuary, China. Marine Chemistry.2010; 119, 52-64.

33. Callahan J, Dai M, Chen RF, Li X, Lu Z, Huang W. Distribution of dissolved organic matter in the Pearl River Estuary, China. Marine Chemistry.2004; 89, 211-224.
