# Supplementary material for: The Spatial and Temporal Distribution of Dissolved Organic Carbon Exported from Three Chinese Rivers to the China Sea
Source: PLoS One. 2016 Oct 18;11(10):e0165039. doi: 10.1371/journal.pone.0165039 (PMC5068779; doi:10.1371/journal.pone.0165039)
Supplement: S3 Table — (DOCX) [file pone.0165039.s003.docx]

S3 Table Compared DOC flux of three rivers in China with the worldwide rivers

| River name (Country) | drainage area (10^6^ km^2^) | Water discharge (10^9^ m^3^ yr^-1^) | DOC flux (10^6^ t yr^-1^) | Reference |
| --- | --- | --- | --- | --- |
| Yenisey (Russia) | 2.58 | 630 | 4.9 | M. Dagg et al., 2004 |
| Mackenzie(Canada) | 1.81 | 310 | 1.3 | M. Dagg et al., 2004 |
| Mississippi (USA) | 3.27 | 530 | 3.5 | M. Dagg et al., 2004 |
| Amazon (Brazil) | 6.15 | 6300 | 19.1 | M. Dagg et al., 2004 |
| Zaire (Zaire) | 3.82 | 1250 | 10.2 | M. Dagg et al., 2004 |
| Orinoco (Venezuela) | 0.99 | 1200 | 4.5 | M. Dagg et al., 2004 |
| Danube | 0.81 | 210 | 0.56 | Cauwet., 2002 |
| Niger (Africa) | 1.21 | 190 | 0.5 | M. Dagg et al., 2004 |
| Pearl River (China) | 0.45 | 350 | 0.82 | This study |
| Yangtze River (China) | 1.8 | 960 | 1.85 | This study |
| Yellow River (China) | 0.75 | 15 | 0.06 | This study |

Reference

Dagg M, R Benner, S Lohrenz, and D Lawrence. Transformation of dissolved and particulate materials on continental shelves influenced by large rivers: Plume processes, Cont. Shelf Re 2004; 24, 833–858, doi:10.1016/j.csr.2004.02.003.

Cauwet G. DOM in the Coastal Zone [M]. In: Hansell, D.A., Carlson, A.C. (Eds.). Biogeochemistry of Marine Dissolved Organic Matter. Academic Press: London, 2002．
