# Supplementary material for: The Spatial and Temporal Distribution of Dissolved Organic Carbon Exported from Three Chinese Rivers to the China Sea
Source: PLoS One. 2016 Oct 18;11(10):e0165039. doi: 10.1371/journal.pone.0165039 (PMC5068779; doi:10.1371/journal.pone.0165039)
Supplement: S4 Table — (DOCX) [file pone.0165039.s004.docx]

**S4 Table Correlation DOC flux/concentration to interaction of temperature and precipitation in regional catchment from three rivers**

In order to determine the correlation the interaction of regional precipitation and temperate indirectly lead to the change of riverine DOC. Based on the model formula: y (ln DOC flux/concentration) = a + b * temperature + c * precipitation + d * temperature * precipitation. Statistical analyses of the results are as follows,

1. **DOC flux in mainstream of the Yangtze River**

**Optimal regression model:** y (ln DOC flux) = 0.023 * temperature + 0.005 * precipitation -2.978 (R^2^ = 0.642)

ANOVA

| Model | Sum of square | df | Mean square | F | Significant |
| --- | --- | --- | --- | --- | --- |
| regression | 16.053 | 2 | 8.027 | 87.953 | 0.001 |
| residual | 8.944 | 98 | 0.091 |  |  |
| total | 24.997 | 100 |  |  |  |

Coefficient

| Model | coefficient | Stand error | Significant |
| --- | --- | --- | --- |
| constant | -2.978 | 0.056 | 0.001 |
| temperature | 0.023 | 0.008 | 0.004** |
| precipitation | 0.005 | 0.001 | 0.001** |

Note: * represent the significant level p < 0.05, **represent the significant level p < 0.01.

1. **DOC flux in tributary of the Yangtze River**

**Optimal regression model:** y (ln DOC flux) = 0.011 * precipitation -10.245 (R^2^ = 0.622)

ANOVA

| Model | Sum of square | df | Mean square | F | Sig. |
| --- | --- | --- | --- | --- | --- |
| regression | 21.396 | 1 | 21.396 | 36.147 | 0.000 |
| residual | 13.022 | 22 | 0.592 |  |  |
| total | 34.418 | 23 |  |  |  |

Coefficient

| Model | coefficient | Stand error | Sig. |
| --- | --- | --- | --- |
| constant | -10.245 | 0.224 | 0.000 |
| precipitation | 0.013 | 0.002 | 0.000** |

Note: * represent the significant level p < 0.05, **represent the significant level p < 0.01.

1. **DOC concentration in tributary of the Yangtze River**

**Optimal regression model:** y (ln DOC concentration) = 0.042 * temperature +1.430 (R^2^ = 0.430)

ANOVA

| Model | Sum of square | df | Mean square | F | Sig. |
| --- | --- | --- | --- | --- | --- |
| regression | 0.779 | 1 | 0.779 | 14.927 | 0.001 |
| residual | 1.149 | 22 | 0.052 |  |  |
| total | 1.928 | 23 |  |  |  |

Coefficient

| Model | coefficient | Stand error | Sig. |
| --- | --- | --- | --- |
| constant | 1.430 | 0.181 | 0.000 |
| temperature | 0.042 | 0.011 | 0.001** |

Note: * represent the significant level p < 0.05, **represent the significant level p < 0.01.

1. **DOC flux in the Yellow River**

**Optimal regression model:** y (ln DOC flux) = 0.036 * precipitation - 0.002 * Temperature * precipitation -5.937 (R^2^ = 0.208)

Parameter Estimates

| Parameter | Estimate | Stand error | 95% confidence interval  Lower bound | 95% confidence interval  Upper bound |
| --- | --- | --- | --- | --- |
| a | -5.937 | 0.151 | -6.238 | -5.637 |
| c | 0.036 | 0.008 | 0.021 | 0.052 |
| d | -0.002 | 0.00 | -0.002 | -0.001 |

ANOVA

| Source | Sum of Squares | df | Mean Squares |
| --- | --- | --- | --- |
| Regression | 2789.486 | 3 | 929.829 |
| Residual | 59.270 | 96 | 0.617 |
| Corrected Total | 74.862 | 98 |  |

Note: “a” represents constant, “c” represents precipitation parameter; “d” represents the parameter of the interaction factors of temperature and precipitation

1. **DOC concentration in the Yellow River**

**Optimal regression model:** y (ln DOC concentration) = 1.138 - 0.015 * precipitation + 0.001 * precipitation * temperature (R^2^ = 0.059)

Parameter Estimates

| Parameter | Estimate | Stand error | 95% confidence interval  Lower bound | 95% confidence interval  Upper bound |
| --- | --- | --- | --- | --- |
| a | 1.138 | 0.117 | 0.906 | 1.370 |
| c | -0.015 | 0.006 | -0.027 | -0.002 |
| d | 0.001 | 0.000 | 0.000 | 0.001 |

ANOVA

| Source | Sum of Squares | df | Mean Squares |
| --- | --- | --- | --- |
| Regression | 94.859 | 3 | 31.620 |
| Residual | 35.409 | 96 | 0.369 |
| Corrected Total | 37.619 | 98 |  |

Note: “a” represents constant, “c” represents precipitation parameter; “d” represents the parameter of the interaction factors of temperature and precipitation

1. **DOC flux in the Pearl River**

**Optimal regression model:** y (ln DOC flux) = - 0.021 * precipitation + 0.001* precipitation * temperature - 4.393 (R^2^ = 0.383)

Parameter Estimates

| Parameter | Estimate | Stand error | 95% confidence interval  Lower bound | 95% confidence interval  Upper bound |
| --- | --- | --- | --- | --- |
| a | -4.393 | 0.199 | -4.789 | -3.997 |
| c | -0.021 | 0.006 | -0.033 | -0.008 |
| d | 0.001 | 0.00 | 0.001 | 0.001 |

ANOVA

| Source | Sum of Squares | df | Mean Squares |
| --- | --- | --- | --- |
| Regression | 1308.621 | 3 | 436.207 |
| Residual | 56.327 | 70 | 0.805 |
| Corrected Total | 91.221 | 72 |  |

Note: “a” represents constant, “c” represents precipitation parameter; “d” represents the parameter of the interaction factors of temperature and precipitation

1. **DOC concentration in the Pearl River**

**Optimal regression model:** y (ln DOC concentration) = 0.304 + 0.001 * precipitation (R^2^ = 0.082)

ANOVA

| Model | Sum of square | df | Mean square | F | Sig. |
| --- | --- | --- | --- | --- | --- |
| regression | 0.607 | 1 | 0.607 | 6.300 | 0.014 |
| residual | 6.839 | 71 | 0.096 |  |  |
| total | 7.446 | 72 |  |  |  |

Coefficient

| Model | coefficient | Stand error | Sig. |
| --- | --- | --- | --- |
| constant | 0.304 | 0.057 | 0.000 |
| precipitation | 0.001 | 0.000 | 0.014** |

Note: * represent the significant level p < 0.05, **represent the significant level p < 0.01.
