# Supplementary figures and images for: The Spatial and Temporal Distribution of Dissolved Organic Carbon Exported from Three Chinese Rivers to the China Sea
Source: PLoS One. 2016 Oct 18;11(10):e0165039. doi: 10.1371/journal.pone.0165039 (PMC5068779; doi:10.1371/journal.pone.0165039)

PRISMA flow diagram


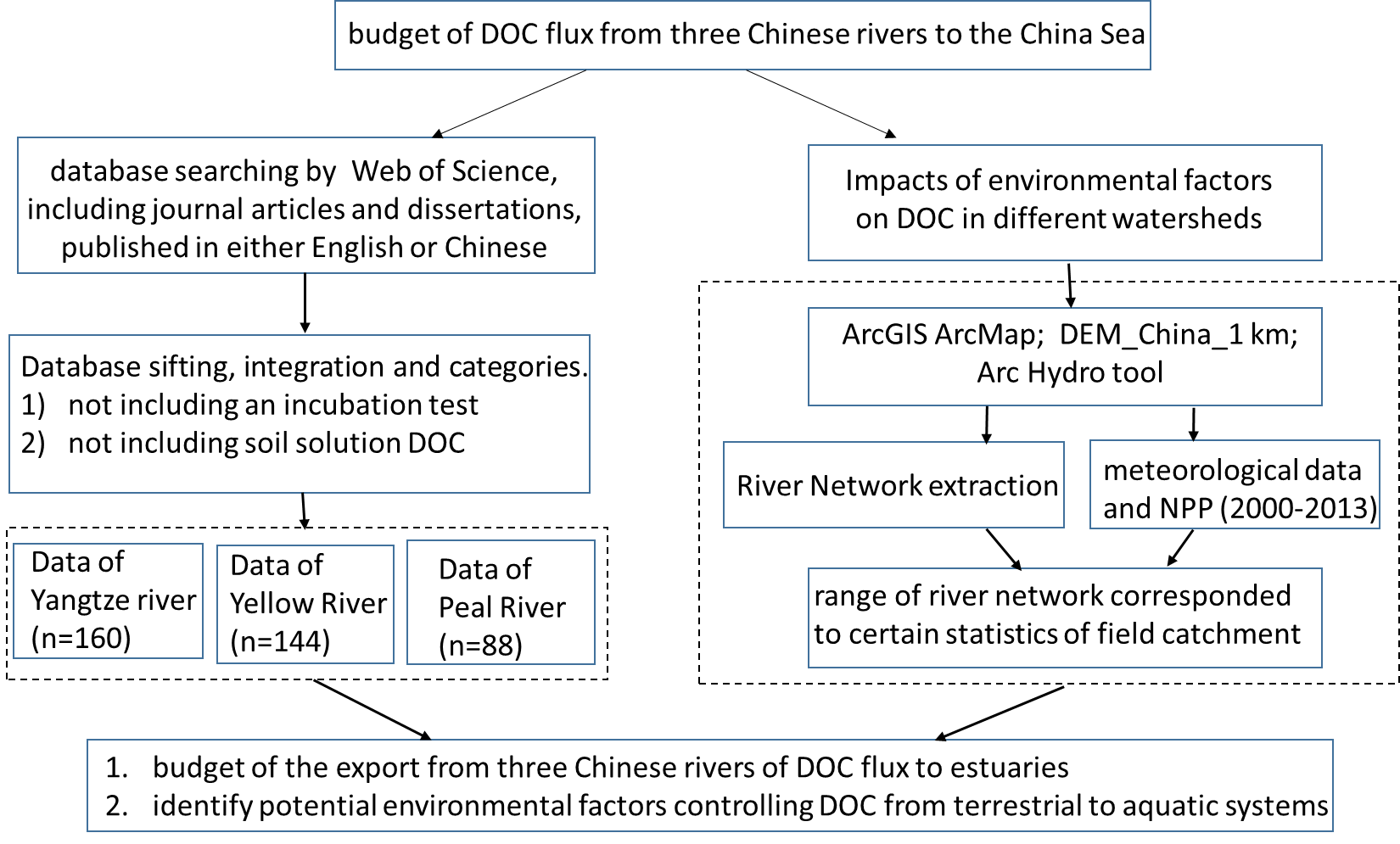

Supplement: S5 Table — (DOC) [file pone.0165039.s005.doc]
